# Genetic evaluation of early-onset atrial fibrillation: impact on patient management

**DOI:** 10.1093/eurheartj/ehaf829

**Published:** 2025-10-30

**Authors:** J Lukas Laws, Mahsima Shabani, Hollie L Williams, Dakota D Grauherr, Wendy M Kilbourne, Diane M Crawford, Isaac Ogunmola, Lili Sun, Zain Virk, Brianna Cathey, Majd A El-Harasis, Cassady J Pelphrey, Joseph A Quintana, Brittany S Murphy, Giovanni E Davogustto, M Edward Ponder, Omeed M Irani, J Michael Daw, Bibin T Varghese, Pablo Saavedra, Robert L Abraham, Juan C Estrada, Katherine T Murray, Walter K Clair, Sharon T Shen, Arvindh N Kanagasundram, Jay A Montgomery, Christopher R Ellis, Frank Fish, Travis D Richardson, George H Crossley, Rebecca R Hung, Jeffrey M Dendy, Adam Wright, Quinn S Wells, Fei Ye, Harikrishna Tandri, William G Stevenson, Megan Lancaster, Prince J Kannankeril, Lynne W Stevenson, Dan M Roden, Zachary T Yoneda, M Benjamin Shoemaker

**Affiliations:** Department of Medicine, Division of Cardiovascular Medicine, Vanderbilt University Medical Center, 1211 Medical Center Drive, Nashville, TN 37232, USA; Department of Medicine, Division of Cardiovascular Medicine, Vanderbilt University Medical Center, 1211 Medical Center Drive, Nashville, TN 37232, USA; Department of Medicine, Division of Cardiovascular Medicine, Vanderbilt University Medical Center, 1211 Medical Center Drive, Nashville, TN 37232, USA; Department of Medicine, Division of Cardiovascular Medicine, Vanderbilt University Medical Center, 1211 Medical Center Drive, Nashville, TN 37232, USA; Department of Medicine, Division of Cardiovascular Medicine, Vanderbilt University Medical Center, 1211 Medical Center Drive, Nashville, TN 37232, USA; Department of Medicine, Division of Cardiovascular Medicine, Vanderbilt University Medical Center, 1211 Medical Center Drive, Nashville, TN 37232, USA; Department of Medicine, Division of Cardiovascular Medicine, Vanderbilt University Medical Center, 1211 Medical Center Drive, Nashville, TN 37232, USA; Department of Biostatistics, Vanderbilt University Medical Center, Nashville, TN, USA; Department of Medicine, Division of Cardiovascular Medicine, Vanderbilt University Medical Center, 1211 Medical Center Drive, Nashville, TN 37232, USA; Department of Medicine, Division of Cardiovascular Medicine, Vanderbilt University Medical Center, 1211 Medical Center Drive, Nashville, TN 37232, USA; Department of Medicine, Division of Cardiovascular Medicine, Vanderbilt University Medical Center, 1211 Medical Center Drive, Nashville, TN 37232, USA; Department of Medicine, Division of Cardiovascular Medicine, Vanderbilt University Medical Center, 1211 Medical Center Drive, Nashville, TN 37232, USA; Department of Medicine, Division of Cardiovascular Medicine, Vanderbilt University Medical Center, 1211 Medical Center Drive, Nashville, TN 37232, USA; Department of Medicine, Division of Cardiovascular Medicine, Vanderbilt University Medical Center, 1211 Medical Center Drive, Nashville, TN 37232, USA; Department of Medicine, Division of Cardiovascular Medicine, Vanderbilt University Medical Center, 1211 Medical Center Drive, Nashville, TN 37232, USA; Department of Medicine, Division of Cardiovascular Medicine, Vanderbilt University Medical Center, 1211 Medical Center Drive, Nashville, TN 37232, USA; Department of Medicine, Division of Cardiovascular Medicine, Vanderbilt University Medical Center, 1211 Medical Center Drive, Nashville, TN 37232, USA; Department of Medicine, Division of Cardiovascular Medicine, Vanderbilt University Medical Center, 1211 Medical Center Drive, Nashville, TN 37232, USA; Department of Medicine, Division of Cardiovascular Medicine, Vanderbilt University Medical Center, 1211 Medical Center Drive, Nashville, TN 37232, USA; Department of Medicine, Division of Cardiovascular Medicine, Vanderbilt University Medical Center, 1211 Medical Center Drive, Nashville, TN 37232, USA; Department of Medicine, Division of Cardiovascular Medicine, Vanderbilt University Medical Center, 1211 Medical Center Drive, Nashville, TN 37232, USA; Department of Medicine, Division of Cardiovascular Medicine, Vanderbilt University Medical Center, 1211 Medical Center Drive, Nashville, TN 37232, USA; Department of Medicine, Division of Cardiovascular Medicine, Vanderbilt University Medical Center, 1211 Medical Center Drive, Nashville, TN 37232, USA; Department of Pharmacology, Vanderbilt University Medical Center, Nashville, TN, USA; Department of Medicine, Division of Cardiovascular Medicine, Vanderbilt University Medical Center, 1211 Medical Center Drive, Nashville, TN 37232, USA; Department of Medicine, Division of Cardiovascular Medicine, Vanderbilt University Medical Center, 1211 Medical Center Drive, Nashville, TN 37232, USA; Department of Medicine, Division of Cardiovascular Medicine, Vanderbilt University Medical Center, 1211 Medical Center Drive, Nashville, TN 37232, USA; Department of Medicine, Division of Cardiovascular Medicine, Vanderbilt University Medical Center, 1211 Medical Center Drive, Nashville, TN 37232, USA; Department of Medicine, Division of Cardiovascular Medicine, Vanderbilt University Medical Center, 1211 Medical Center Drive, Nashville, TN 37232, USA; Department of Pediatrics, Vanderbilt University Medical Center, Nashville, TN, USA; Department of Medicine, Division of Cardiovascular Medicine, Vanderbilt University Medical Center, 1211 Medical Center Drive, Nashville, TN 37232, USA; Department of Medicine, Division of Cardiovascular Medicine, Vanderbilt University Medical Center, 1211 Medical Center Drive, Nashville, TN 37232, USA; Department of Medicine, Division of Cardiovascular Medicine, Vanderbilt University Medical Center, 1211 Medical Center Drive, Nashville, TN 37232, USA; Department of Medicine, Division of Cardiovascular Medicine, Vanderbilt University Medical Center, 1211 Medical Center Drive, Nashville, TN 37232, USA; Department of Biomedical Informatics, Vanderbilt University Medical Center, Nashville, TN, USA; Department of Medicine, Division of Cardiovascular Medicine, Vanderbilt University Medical Center, 1211 Medical Center Drive, Nashville, TN 37232, USA; Department of Pharmacology, Vanderbilt University Medical Center, Nashville, TN, USA; Department of Biomedical Informatics, Vanderbilt University Medical Center, Nashville, TN, USA; Department of Medicine, Division of Cardiovascular Medicine, Vanderbilt University Medical Center, 1211 Medical Center Drive, Nashville, TN 37232, USA; Department of Biostatistics, Vanderbilt University Medical Center, Nashville, TN, USA; Department of Medicine, Division of Cardiovascular Medicine, Vanderbilt University Medical Center, 1211 Medical Center Drive, Nashville, TN 37232, USA; Department of Medicine, Division of Cardiovascular Medicine, Vanderbilt University Medical Center, 1211 Medical Center Drive, Nashville, TN 37232, USA; Department of Medicine, Division of Cardiovascular Medicine, Vanderbilt University Medical Center, 1211 Medical Center Drive, Nashville, TN 37232, USA; Department of Pediatrics, Vanderbilt University Medical Center, Nashville, TN, USA; Department of Medicine, Division of Cardiovascular Medicine, Vanderbilt University Medical Center, 1211 Medical Center Drive, Nashville, TN 37232, USA; Department of Medicine, Division of Cardiovascular Medicine, Vanderbilt University Medical Center, 1211 Medical Center Drive, Nashville, TN 37232, USA; Department of Pharmacology, Vanderbilt University Medical Center, Nashville, TN, USA; Department of Biomedical Informatics, Vanderbilt University Medical Center, Nashville, TN, USA; Department of Medicine, Division of Cardiovascular Medicine, Vanderbilt University Medical Center, 1211 Medical Center Drive, Nashville, TN 37232, USA; Department of Medicine, Division of Cardiovascular Medicine, Vanderbilt University Medical Center, 1211 Medical Center Drive, Nashville, TN 37232, USA

**Keywords:** Atrial fibrillation, Genetic testing, Dilated cardiomyopathy, Hypertrophic cardiomyopathy, Arrhythmogenic cardiomyopathy

## Abstract

**Background and Aims:**

Genetic testing is recommended for select patients with atrial fibrillation (AF). The aims of this study were to define the results of genetic evaluation and its therapeutic impact for patients referred to a dedicated AF precision medicine clinic.

**Methods:**

Patients diagnosed with AF before age 60 were candidates for referral. In addition to standard evaluation with history, physical exam, and electrocardiogram (ECG), genetic evaluation included a three-generation pedigree, cardiac imaging, ambulatory monitoring, and clinical genetic testing with a cardiomyopathy/arrhythmia panel.

**Results:**

Overall, 264 participants were referred: the median age was 47 years (Q1, Q3: 38, 55), 77 (29%) were female, and 236 (89%) were White. Median age at AF diagnosis was 39 years (Q1, Q3: 31, 48), and median time from AF diagnosis to evaluation was 3.7 years (Q1, Q3: 0.9, 10). A total of 242 patients (92%) underwent genetic testing, which identified a pathogenic or likely pathogenic variant in 48 (20%). The strongest predictors of positive genetic testing were history of cardiomyopathy, infranodal conduction disease, and elevated T1 or late gadolinium enhancement on cardiac magnetic resonance imaging (all *P* < .05). The strongest predictors of negative genetic testing were obstructive sleep apnoea and a normal 12-lead ECG (both *P* < .04). Overall, genetic testing changed clinical management in 52% of patients with positive genetic testing, highlighted by seven new implantable cardioverter-defibrillator placements and initiation of disease-modifying therapy in 16 patients.

**Conclusions:**

Genetic testing was positive in 20% of patients with early-onset AF referred to a dedicated AF precision medicine clinic. Genetic testing results may change clinical management in genotype-positive patients.

## Structured Graphical Abstract

**Structured Graphical Abstract:**
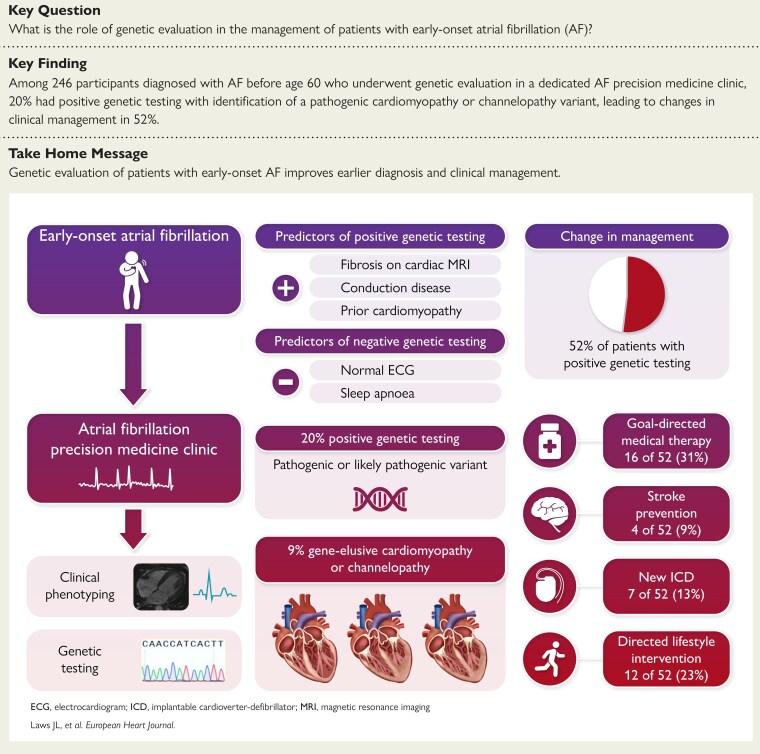
Genetic evaluation of patients with early onset atrial fibrillation in a dedicated precision medicine clinic demonstrated high yield of pathogenic variants and significant impact on clinical management. ECG: electrocardiogram, ICD: implantable cardiac-defibrillator, MRI: magnetic resonance imaging.

**See the editorial comment for this article ‘Genetic testing in cardiology: insights into ‘overlap' from atrial fibrillation’, by C.A. MacRae, https://doi.org/10.1093/eurheartj/ehaf988.**

## Introduction

Atrial fibrillation (AF) is estimated to affect over 30 million people worldwide with most patients diagnosed at an advanced age.^1^ Atrial fibrillation was not historically considered a genetic syndrome until case reports began emerging of patients and families with early-onset AF and genetic ‘overlap syndromes’. These patients presented initially with AF and later developed other serious cardiac manifestations such as heart failure, conduction disease, or sudden death due to rare genetic variants. Early examples included syndromes associated with pathogenic variants in *KCNQ1* [long or short QT syndrome (LQTS/SQTS)], *SCN5A* [Brugada syndrome (BrS), progressive cardiac conduction disease (PCCD)], and *LMNA* [arrhythmogenic cardiomyopathy (ACM)].^2–4^ At the time, these cases were thought to be extremely rare, so genetic testing was not recommended.^5^ That idea was challenged in 2018 with results from large sequencing studies that identified an association between pathogenic variants in *TTN* and early-onset AF.^6,7^  *TTN* is the leading monogenic cause of dilated cardiomyopathy (DCM), and pathogenic, truncating variants in *TTN* were found in 6.5% of patients with AF diagnosed before age 30% and 2.1% of patients diagnosed before age 65.^6^ These results raised the question of whether clinical genetic testing should be considered for patients with early-onset AF.^8,9^ Accordingly, a study in 2021 analysed 145 genes that were included on clinical cardiomyopathy and arrhythmia gene panels and found that in patients with AF diagnosed before age 65, the prevalence of positive genetic testing [identification of a pathogenic or likely pathogenic (P/LP) variant] was up to 10.1%.^10^

Practice guidelines have incorporated this new evidence and now support the use of genetic testing for select patients with AF.^11,12^ The recommendations state that the benefit of genetic testing in AF is to inform prognosis, which is supported by results demonstrating a significantly higher risk of mortality for patients with AF who possess a pathogenic cardiomyopathy or arrhythmia variant.^11–13^ The increased mortality risk associated with these variants is likely not from AF directly, but instead from development of an overlapping cardiomyopathy or arrhythmia syndrome that can lead to fatal ventricular arrhythmias and/or heart failure [e.g. ACM, DCM, or hypertrophic cardiomyopathy (HCM)].^14,15^ A major knowledge gap we seek to address is whether genetic testing for patients with early-onset AF can be used to guide therapeutic decision-making by facilitating the diagnosis of an AF overlap syndrome. Accordingly, we hypothesized that a detailed evaluation in a dedicated AF precision medicine clinic consisting of genetic testing and detailed phenotyping would have important diagnostic and therapeutic implications for patients with early-onset AF.

## Methods

### Genetic evaluation

**Figure 1:**
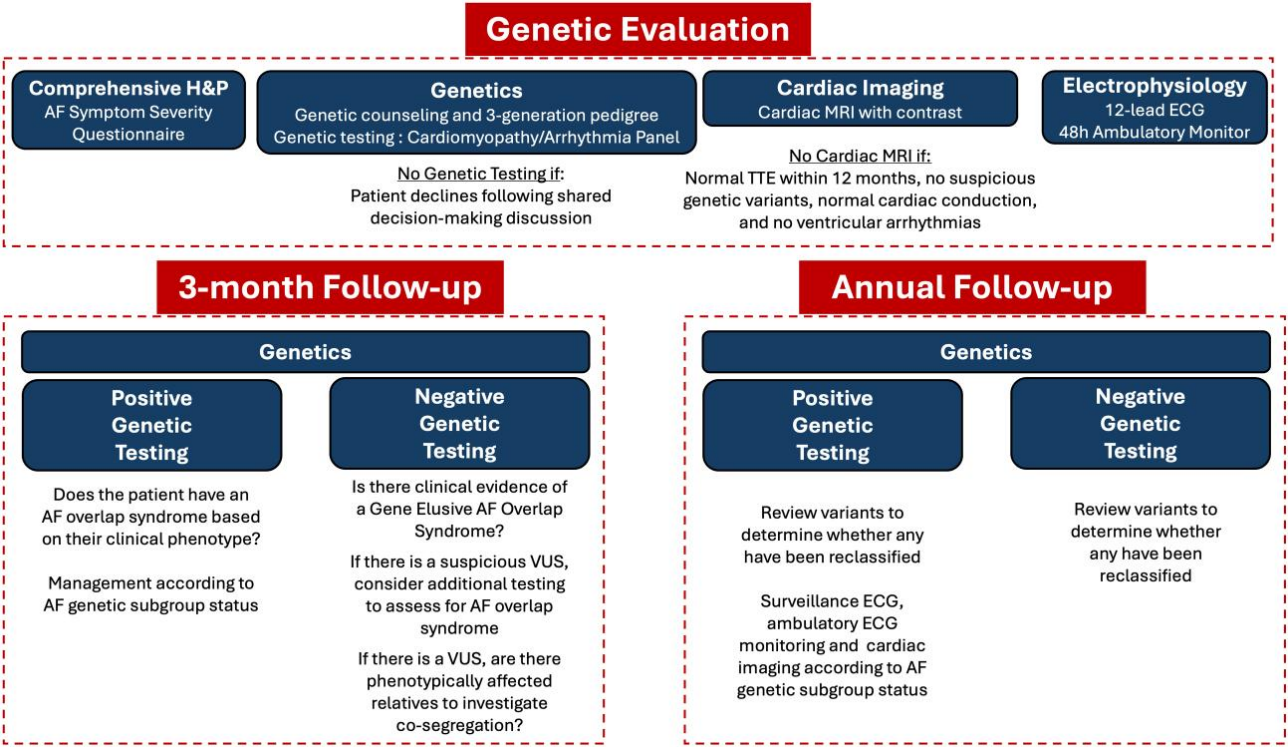
Framework for evaluation in the atrial fibrillation precision medicine clinic. Comprehensive evaluation includes an assessment of both genetic predisposition and clinical phenotyping to evaluate all cardiac manifestations of genetic disease

Participants were referred for genetic evaluation of AF between October 2020 and September 2024. The AF Precision Medicine Clinic, which was formally established in April 2023, sees more than 350 new patients per year. Clinical providers could refer based on clinical suspicion for a genetic aetiology or were prompted to refer young patients with AF via an automated Best Practice Alert through the electronic medical record (see Supplementary data online, *Figures S1*–*S3*). Genetic evaluation consisted of both genetic testing and clinical phenotyping to evaluate for AF overlap syndromes that may be present with or without positive genetic testing. Patients with cardiomyopathy or ventricular arrhythmias were eligible for enrolment in the registry only if AF was diagnosed prior to cardiomyopathy or if cardiomyopathy was identified concurrently as part of the diagnostic workup of AF. *Figure 1* shows the framework for genetic evaluation. Briefly, patients diagnosed with AF prior to 60 years of age and/or those with a strong family history of AF were candidates for referral based on a suspicion for genetic susceptibility to AF. The initial clinic visit consisted of a comprehensive history and physical examination, a baseline AF symptom severity questionnaire, 12-lead electrocardiogram (ECG), construction of a three-generation pedigree, and genetic counselling. Standard testing included a continuous ambulatory ECG monitor and cardiac imaging [cardiac magnetic resonance imaging (MRI) or transthoracic echocardiography]. Most patients underwent clinical genetic testing with a comprehensive cardiomyopathy and arrhythmia panel using commercial vendors (Labcorp/Invitae, Burlington, NC; GeneDx, Stamford, CT) or the CLIA-approved Vanderbilt University Medical Center Clinical Genetic Testing Laboratory (Nashville, TN). Some family members underwent targeted genotyping limited to only the familial variant. Depending on the results of the standard clinical evaluation and genetic testing, additional diagnostic testing with an exercise tolerance test, extended ECG monitoring, or a procainamide challenge was performed. Patients were seen for a 3-month follow-up appointment either in-person or by telemedicine to review the results of their tests and develop a longitudinal care plan.

### Variant interpretation

The clinical genetic testing laboratories for this study used the ACMG/AMP (American College of Medical Genetics and Genomics and Association for Molecular Pathology) standards and guidelines for rare variant interpretation accounting for population prevalence using ancestry-stratified minor allele frequencies.^16^ Positive genetic testing was defined as the presence of one or more P/LP variants in a gene with autosomal dominant (AD) inheritance, or two P/LP variants in a gene with autosomal recessive (AR) inheritance. For genes with X-linked inheritance, genetic testing was considered positive for hemizygous men or homozygous women. Due to variable splicing in cardiac and skeletal myocytes, P/LP *TTN* variants were required to be in a cardiac exon with a proportion spliced index ≥ 90% to be classified as positive.^17,18^ All patients whose genetic testing results did not meet the positive genetic testing criteria were considered to have negative genetic testing. Negative genetic testing included those with variants of undetermined significance (VUS), heterozygous carriers of variants in a gene with AR inheritance, and patients with no rare variants reported. Some VUS were designated as suspicious if they (1) possessed characteristics that suggested a potential pathogenic role but lacked sufficient evidence to be definitively classified as P/LP,^19^ (2) were in a strong/definitive evidence gene, and (3) were potentially consistent with the clinical phenotype of the participant.

### Atrial fibrillation genetic subgroups

Genes on the cardiomyopathy and arrhythmia genetic testing panel are included because of previously reported association with an inherited cardiomyopathy or arrhythmia syndrome. Genes were classified according to the predominant gene-phenotype association here termed ‘AF genetic subgroups’. *Table 1* lists the AF genetic subgroups and corresponding genes, and the full gene panel used for evaluation is listed in Supplementary data online, *Table S1*. For this analysis, the AF genetic subgroups are limited to DCM genes, ACM genes, HCM genes, and channelopathy genes. While ACM is a broader, non-specific term, it is used here to classify both arrhythmogenic right ventricular cardiomyopathy (ARVC) and non-dilated left ventricular cardiomyopathy (NDLVC) due to the significant overlap of shared phenotypic features and genetic predisposition between these two diagnoses.^20^ Additional detailed methods regarding enrolment, variant interpretation, and phenotype classification are reported in Supplementary data online, *Methods S1*.

**Table 1:** Major genes listed according to proposed atrial fibrillation genetic subgroups

DCM	ACM (ARVC, NDLVC)	HCM	Channelopathies (Brugada, LQTS/SQTS, CPVT, PCCD)
*TTN*	*DSP*	*MYBPC3*	*KCNQ1*
	*EMD* (XL)	*MYH7*	*KCNH2*
	*FLNC*	*ACTC1*	*SCN5A*
	*LMNA*	*ACTN2*	*RYR2*
	*PKP2*	*ALPK3*	*CACNA1C*
	*TRPM4*	*CSRP3*	*CALM1*
	*BAG3*	*MYL2*	*CALM2*
	*DES*	*MYL3*	*CALM3*
	*DSC2*	*PRKAG2*	*CASQ2*
	*DSG2*	*TNNC1*	*HCN4*
	*JUP*	*TNNI3*	*KCNE1*
	*PLN*	*TNNT2*	*KCNJ2*
	*RBM20*	*TPM1*	*TRDN* (AR)
	*TMEM43*		

Genes are listed by their predominant associated phenotype. Genes with definitive or strong evidence for gene–disease validity or clinical actionability are listed here, plus *KCNE1* and *KCNJ2* were added based on newer evidence. A complete list of genes used in the comprehensive panel can be found in Supplementary data online, *Table S1*.

ACM, arrhythmogenic cardiomyopathy; ARVC, arrhythmogenic right ventricular cardiomyopathy; NDLVC, non-dilated left ventricular cardiomyopathy; LQTS, long QT syndrome; CPVT, catecholaminergic polymorphic VT; PCCD, progressive cardiac conduction disease; SQTS, short QT syndrome; XL, X-linked inheritance; AR, autosomal recessive inheritance.

### Penetrance of the ventricular phenotype and management considerations

**Figure 2:**
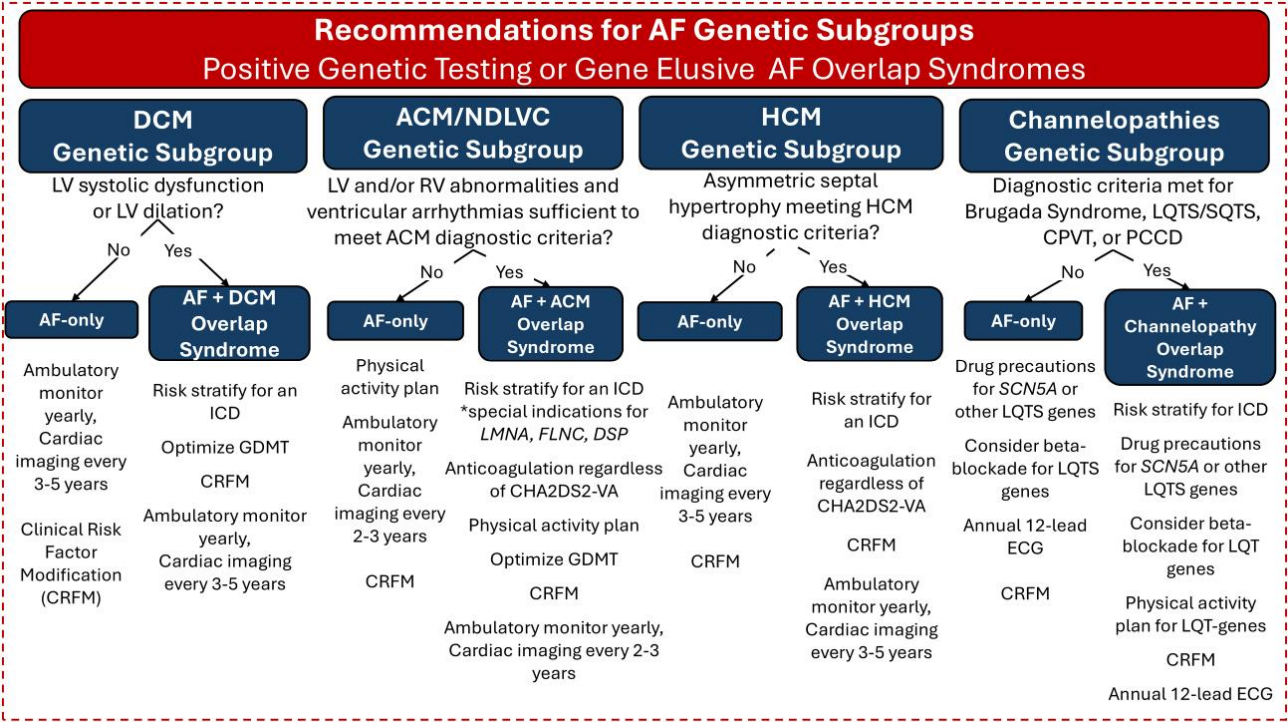
Management recommendations for the atrial fibrillation genetic subgroups. Classification of patients with positive genetic testing using genotype association with risk for atrial fibrillation overlap syndromes facilitates individualized surveillance and management changes

Patients with AF and positive genetic testing may or may not develop any clinical manifestations of the AF overlap syndrome for which they are genetically susceptible. Official diagnostic criteria defined by practice guidelines were used for DCM, ACM, HCM, BrS, and LQTS/SQTS, catecholaminergic polymorphic ventricular tachycardia, and PCCD (see Supplementary data online, *Table S2*). Patients with AF who also met the diagnostic criteria for an overlapping cardiomyopathy or arrhythmia syndrome were designated as having an ‘AF overlap syndrome’ with management considerations shown in *Figure 2*. Patients with AF who did not meet diagnostic criteria for their corresponding overlap syndrome were designated as ‘AF-only’. In this study, all the patients with positive genetic testing could be considered to have penetrant disease with respect to AF, but disease penetrance is primarily used to refer to the presence or absence of the corresponding cardiomyopathy or arrhythmia overlap syndrome.

All the syndromes evaluated are also known to occur in the absence of an identifiable genetic variant. For this analysis, the term used is ‘gene elusive’ but elsewhere can also be termed ‘genotype-negative’. Historically, the rate of gene elusive DCM is estimated to be 50%–80%, ACM is 40%–50%, HCM is 40%, LQTS is 15%–30%, and BrS is 80%–90%.^12,21–24^

For patients with positive genetic testing or gene elusive AF overlap syndromes, changes in clinical management occurred according to guidelines that exist for the specific overlap syndrome and are presented in *Figure 2* and Supplementary data online, *Table S2*.

### Statistical analysis

Descriptive statistics for enrolment demographics and characteristics, genetic testing results, and phenotypic evaluation are reported for probands that completed the evaluation (both clinical genetic testing and phenotyping). Penetrance of the ventricular phenotype and changes to clinical management are reported for all patients that completed genetic testing and phenotypic evaluation, including relatives identified through cascade screening and targeted single variant genetic testing. Patients were grouped by positive genetic testing, gene elusive AF overlap syndrome, or negative genetic evaluation. Univariate analysis of group differences was determined by χ² test or Fisher’s exact test for categorical variables with low expected counts and Kruskal–Wallis or pairwise Wilcoxon rank-sum tests for continuous variables. Logistic regression estimated the association between pre-specified clinical variables and positive genetic testing. Each variable was modelled separately with adjustment for age at AF diagnosis, sex, and the diagnosis-to-testing interval (key covariates chosen *a priori* to address age-related penetrance of associated overlap syndromes). Limited sample size precluded a single multivariable model including all candidate predictors and confounders. Confidence intervals and hypothesis testing thresholds were not adjusted for multiple testing given the exploratory and hypothesis-generating goal of this analysis. All statistical analysis was performed using R statistical software version 4.4.2 (R Foundation for Statistical Computing, Vienna, Austria).

## Results

### Yield of genetic testing and phenotypic evaluation

**Figure 3:**
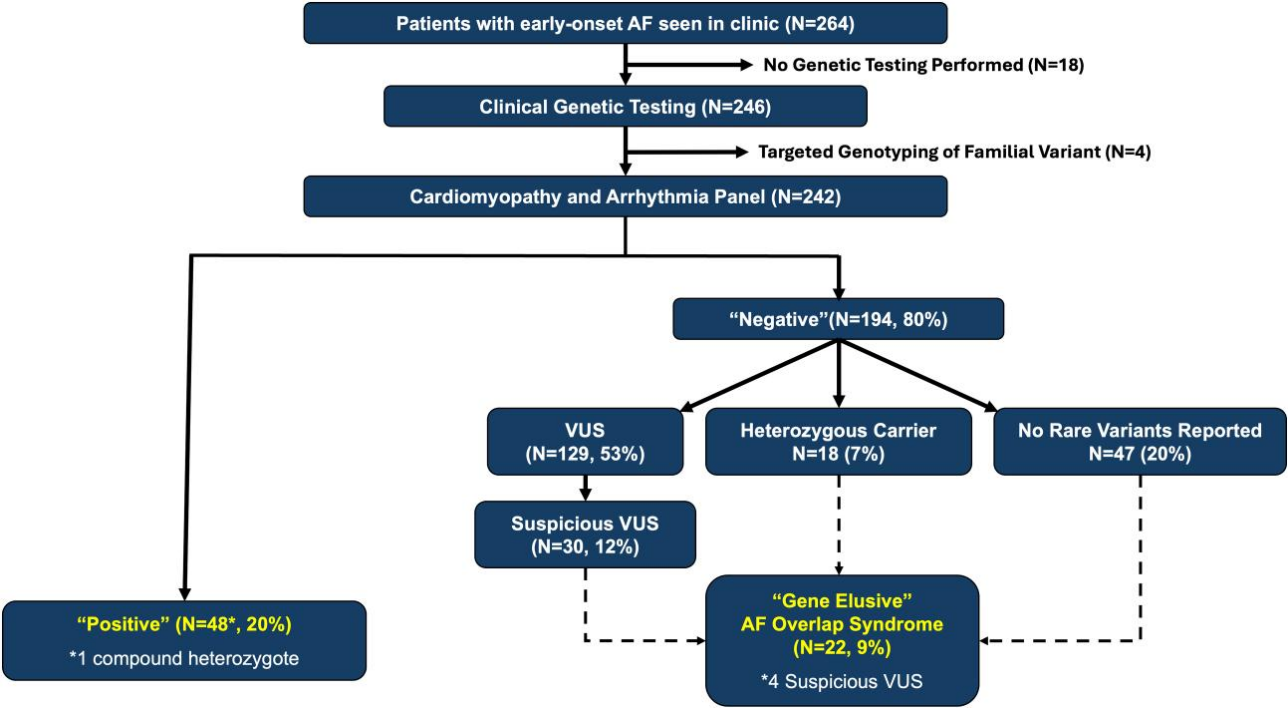
Genetic testing results from the atrial fibrillation precision medicine clinic

**Figure 4:**
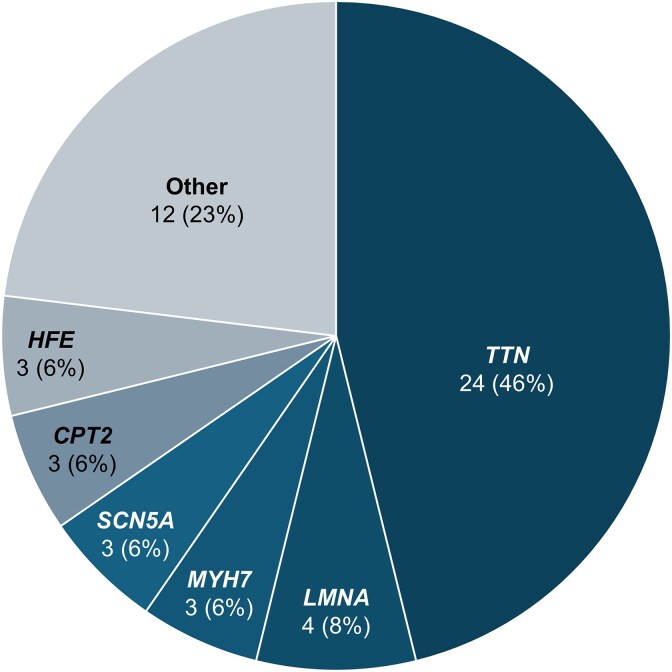
Positive genetic testing results according to gene. Pathogenic or likely pathogenic *TTN* variants were the most prevalent of patients with positive genetic testing. Other genes with strong association with cardiomyopathy *LMNA* and *MYH7* were also prevalent, in addition to the channelopathy gene *SCN5A* associated with Brugada syndrome. Variants in *LMNA* and *MYH7* were the other genes that comprised the largest individual patient pools, with other individual genes less commonly discovered in this cohort. *Percentages sum to 101% due to rounding error

**Figure 5:**
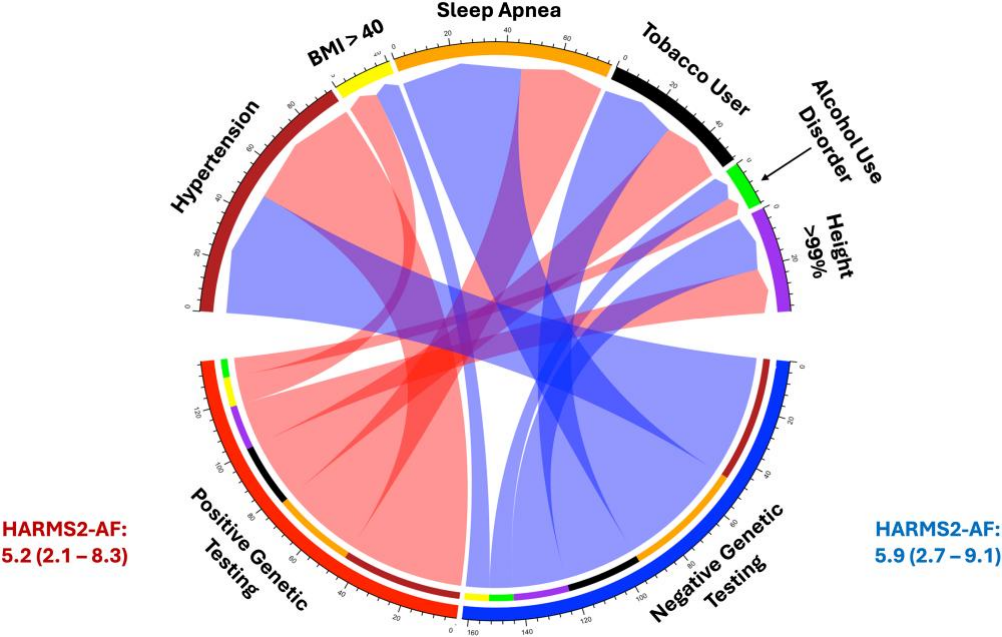
Chord diagram illustrates that patients with major clinical risk factors for atrial fibrillation have positive genetic testing. HARMS2-AF is a clinical risk score that found no difference in aggregated clinical risk estimates between patients with positive vs negative genetic testing (*P* = .76)

Overall, 264 patients were referred for genetic evaluation and prospectively enrolled (*Figure 3*). The median age at time of evaluation was 47 years and the median age at AF diagnosis was 39 years. Genetic evaluation was completed in 242 probands (*Table 2*). Full details of clinical genetic testing are included in Supplementary data online, *Table S3*. Genetic testing was positive in 48 (20%) of unrelated probands (*Figure 4* and *Figure 5*), including 1 that was a compound heterozygote (*Table 3*). A diagnosis of a cardiomyopathy or ventricular arrhythmia syndrome had been made in 6 of 48 probands prior to referral to the clinic for genetic evaluation. An additional four variant carriers, each from separate families, were identified through targeted cascade screening. Among participants with negative genetic testing, 129 participants (53%) had a VUS and 30 participants (12%) had a VUS that was considered suspicious (see Supplementary data online, *Table S4*). Among the remaining participants, 18 participants (7%) were heterozygous carriers of a P/LP variant for an AR syndrome and 47 participants (20%) had no rare variants reported. Among the participants with negative genetic testing, clinical testing results identified a gene elusive AF overlap syndrome in 22 (9%) (*Figure 3* and *Table 4*).

**Table 2:** Baseline demographics and clinical characteristics of patients at time of referral to the AF Precision Medicine Clinic

	Total cohort (*N* = 242)	Genetic evaluation groups		
		Positive genetic testing (*N* = 48)	Gene elusive AF overlap syndrome (*N* = 22)	Negative genetic evaluation (*N* = 172)
Demographics				
Age at enrolment (years)	46 (12)	49 (12)	46 (11)	46 (12)
Age at AF diagnosis (years)	39 (11)	37 (12)	39 (12)	40 (11)
Sex (Male)	168 (69%)	34 (71%)	18 (82%)	116 (67%)
Race				
White	215 (89%)	41 (85%)	17 (77%)	157 (91%)
Black	21 (9%)	4 (8%)	5 (23%)	12 (7%)
American Indian/Alaska Native	1 (0%)	0 (0%)	0 (0%)	1 (0.6%)
Asian	5 (2%)	3 (6%)	0 (0%)	2 (1%)
Ethnicity				
Not Hispanic	234 (97%)	46 (96%)	22 (100%)	166 (97%)
Hispanic	8 (3%)	2 (4%)	0 (0%)	6 (4%)
Height (cm)	178 (11)	175 (10)	181 (10)	178 (11)
Weight (kg)	100 (24)	96 (23)	101 (22)	101 (24)
BMI (kg/m^2^)	31 [27, 35]	29 [27, 34]	30.6 [28, 32]	30.9 [28, 36]
Medical history				
Hypertension	115 (48%)	21 (44%)	11 (50%)	83 (48%)
Coronary artery disease	22 (9%)	5 (10%)	6 (27%)	11 (6%)
Prior myocardial infarction	5 (2%)	0 (0%)	2 (9%)	3 (2%)
Prior stroke or transient ischaemic attack	9 (4%)	3 (6%)	0 (0%)	6 (3.5%)
Valvular heart disease	1 (0%)	0 (0%)	0 (0%)	1 (0.6%)
Diabetes mellitus	26 (11%)	8 (17%)	2 (9%)	16 (9%)
Obstructive sleep apnoea	101 (42%)	14 (29%)	8 (36%)	79 (46%)
Thyroid disorder (hyper or hypothyroid)	24 (10%)	5 (10%)	1 (5%)	18 (11%)
Smoking, vaping, or other tobacco history	66 (27%)	12 (25%)	5 (23%)	49 (29%)
Alcohol use disorder	21 (9%)	3 (6%)	2 (9%)	16 (9%)
Illicit drug use	12 (5%)	1 (2%)	3 (14%)	8 (5%)
Physical endurance training	38 (16%)	6 (13%)	7 (32%)	25 (15%)
Familial atrial fibrillation	108 (45%)	22 (46%)	9 (41%)	77 (45%)
Syncope	30 (12%)	4 (8%)	2 (9%)	24 (14%)
Clinical characteristics at enrolment				
Type of AF				
Paroxysmal	180 (74%)	34 (71%)	11 (50%)	135 (79%)
Persistent	60 (25%)	13 (27%)	11 (50%)	36 (21%)
Long standing persistent	2 (0.8%)	1 (2%)	0 (0%)	1 (0.6%)
Other atrial arrhythmias (flutter, AVNRT, undefined SVT)	57 (24%)	9 (19%)	5 (23%)	42 (24%)
Number of cardioversions^a^	0 [0, 1]	0.5 [0, 2]	2 [1, 2]	0 [0, 1]
CHA2DS2-VA score	1 [0, 2]	1 [0, 2]	1 [0, 2]	1 [0, 1]
AF ablation	104 (43%)	21 (44%)	11 (50%)	72 (42%)
Permanent pacemaker	10 (4%)	3 (6%)	1 (5%)	6 (4%)
History of LV systolic dysfunction	38 (16%)	13 (27%)	13 (59%)	12 (7%)
Implantable cardiac defibrillator		8 (17%)	4 (18%)	2 (1%)
Primary prevention	8 (3%)	4 (8%)	2 (9%)	2 (1%)
Secondary prevention	6 (2%)	4 (8%)	2 (9%)	0 (0%)
Ventricular arrhythmias				
None	169 (70%)	29 (60%)	12 (55%)	128 (74%)
Premature ventricular contractions (PVCs)	47 (19%)	9 (19%)	7 (32%)	31 (18%)
Complex ventricular ectopy (couplets, NSVT)	22 (9%)	7 (15%)	3 (14%)	12 (7%)
Sustained ventricular tachycardia/fibrillation	4 (2%)	3 (6%)	0 (0%)	1 (0.6%)
Time from AF diagnosis to genetic testing (years)	3.8 [1, 10]	6.6 [3, 20]	2.5 [0.8, 8]	35 [0.8, 8]

Continuous variables are listed as median [IQR] or mean (SD), and categorical variables are listed as number (percentage). Data include only probands who underwent genetic testing with a full cardiomyopathy and arrhythmia panel (*N* = 242; *Figure 1*).

^a^Both electrical and pharmacologic cardioversions.

**Table 3:** Variants in probands with positive genetic testing

Patient	Gene	Inheritance pattern	Predominant overlap syndrome for gene	c.DNA	p. Amino Acid	Variant class
1	*TTN*	AD	DCM	c.56572C>T	p.Arg18858*	Likely pathogenic
2	*TTN*	AD	DCM	c.3642T>A	p.Tyr1214*	Likely pathogenic
3	*TTN*	AD	DCM	c.70162C>T	p.Arg23388*	Pathogenic
4	*TTN*	AD	DCM	c.100587G>A	p.Trp33529*	Likely pathogenic
5	*TTN*	AD	DCM	c.12587C>A	p.Ser4196*	Likely pathogenic
6	*TTN*	AD	DCM	c.56572C>T	p.Arg18858*	Likely pathogenic
7	*TTN*	AD	DCM	c.45316_45320dup	p.Arg15108Alafs*71	Pathogenic
8	*TTN*	AD	DCM	c.98299_98300del	p.Arg32767Glyfs*2	Likely pathogenic
9	*TTN*	AD	DCM	c.73031G>A	p.Trp24344*	Likely pathogenic
10	*TTN*	AD	DCM	c.9448C>T	p.Arg3150*	Likely pathogenic
11	*TTN*	AD	DCM	c.54120del	p.Gly18041Alafs*44	Likely pathogenic
12	*TTN*	AD	DCM	c.94263del	p.Ala31422Profs*35	Likely pathogenic
13	*TTN*	AD	DCM	c.67095_67908dupTCTT	p.Thr22637Serfs*3	Likely pathogenic
14	*TTN*	AD	DCM	c.67833C>G	p.Tyr22611*	Likely pathogenic
15	*TTN*	AD	DCM	c.53599G>T	p.Glu17867*	Likely pathogenic
16	*TTN*	AD	DCM	c.107223+2T>C	Intronic	Likely pathogenic
17	*TTN*	AD	DCM	c.42014_42021del	p.Arg14005Thrfs*4	Likely pathogenic
18	*TTN*	AD	DCM	c.49870C>T	p.Arg16624*	Likely pathogenic
19	*TTN*	AD	DCM	c.58732+2T>C	Intronic	Likely pathogenic
20	*TTN*	AD	DCM	c.77145dup	p.Ser25716Leufs*8	Likely pathogenic
21	*TTN*	AD	DCM	c.57603C>A	p.Cys19201*	Likely pathogenic
22	*TTN*	AD	DCM	c.48167del	p.Pro16056Glnfs*7	Likely pathogenic
23	*TTN*	AD	DCM	c.63370C>T	p.Gln21124*	Likely pathogenic
24	*LMNA*	AD	ACM	c.658C>T	p.Arg220Cys	Likely pathogenic
25	*LMNA*	AD	ACM	c.647G>A	p.Arg216His	Likely pathogenic
26	*LMNA*	AD	ACM	c.1146C>T	p.Gly382=	Likely pathogenic
27	*PKP2*	AD	ACM	c.1237C>T	p.Arg413*	Pathogenic
28	*FLNC*	AD	ACM	c.970-4A>G	Intronic	Pathogenic
29	*EMD*	AD	ACM	Complete Deletion	Complete Deletion	Pathogenic
30	*TRPM4*	AD	ACM	c.3119T>C	p.Iso1040Thr	Pathogenic
31*	*DSP*	AD	ACM	c.5212C>T	p.Arg1738*	Pathogenic
32	*MYH7*	AD	HCM	c.4348G>A	p.Asp1450Asn	Pathogenic
33	*MYH7*	AD	HCM	c.1988G>A	p.Arg663His	Pathogenic
34	*MYBPC3*	AD	HCM	c.833delG	p.Gly278Glufs*22	Pathogenic
35	*SCN5A*	AD	Channelopathy	c.2369T>A	p.Ile790Asn	Likely pathogenic
36	*SCN5A*	AD	Channelopathy	c.393-2A>G	Intronic	Likely pathogenic
37	*SCN5A*	AD	Channelopathy	c.5126C>T	p.Thr1709Met	Likely pathogenic
38	*KCNQ1*	AD	Channelopathy	c.564G>A	p.Trp188*	Pathogenic
31*	*HFE*	AR	Hemochromatosis	c.845G>A/c.187C>G	p.Cys282Tyr/p.His63Asp	Pathogenic
39	*HFE*	AR	Hemochromatosis	c.845G>A/c.187C>G	p.Cys282Tyr/p.His63Asp	Pathogenic
40	*HFE*	AR	Hemochromatosis	c.845G>A/c.187C>G	p.Cys282Tyr/p.His63Asp	Pathogenic
41	*HFE*	AR	Hemochromatosis	c.845G>A/c.187C>G	p.Cys282Tyr/p.His63Asp	Pathogenic
42	*CPT2*	AD/AR	CPT2 deficiency	c.370C>T	p.Arg124*	Pathogenic
43	*CPT2*	AD/AR	CPT2 deficiency	c.691C>T	p.Arg231Trp	Likely pathogenic
44	*CPT2*	AD/AR	CPT2 deficiency	c.338C>T	p.Ser113Leu	Pathogenic
45	*TTR*	AD	TTR amyloidosis	c.424G>A	p.Val142Ile	Pathogenic
46	*TTR*	AD	TTR amyloidosis	c.424G>A	p.Val142Ile	Pathogenic
47	*LZTR1*	AD/AR	Noonan syndrome	c.485G>A	p.Trp162*	Pathogenic
48	*GJA5*	AD	Familial AF	Complete deletion	Complete deletion	Pathogenic

* = premature protein termination.

**Table 4:** Participants with gene elusive atrial fibrillation overlap syndromes (*N* = 22)

	Diagnosis	Age range at AF diagnosis (years)	Ventricular structure and function	Ventricular arrhythmias	Sinus node and conduction	Family history	Genetic testing
1	ACM/NDLVC	56–60	Non-dilated LV with hypokinesis Worst LVEF 10%, now 50% Septal intramural LGE	Non-sustained VT Negative EP study	SSS requiring PPM, LBBB	Negative	Negative, suspicious VUS in *ACTC1* c.309C>A
2	ACM/NDLVC	21–25	Non-dilated LV with normal function RV dilation and hypokinesis (RVEF 40%) No LGE	Non-sustained VT	Normal	SCD ICD	Negative, VUS × 1
3	ACM/NDLVC	36–40	Non-dilated chambers with biventricular systolic dysfunction (LVEF 38%)	Non-sustained VT	Normal	SCD	Negative, no rare variants
4	ACM/NDLVC	31–35	Non-dilated LV/RV with hypokinesis LVEF 47% Ring-like intramural LGE	PVCs>500/day	IVCD	Familial AF	Negative, VUS × 1
5	ACM/NDLVC	56–60	Non-dilated LV with hypokinesis Worst LVEF 30%, now 65% MRI uninterpretable	Ventricular couplets	Normal	Familial AF, cardiomyopathy	Negative, VUS × 1
6	ACM/NDLVC	46–50	Non-dilated LV with hypokinesis Worst LVEF 40%, now 57% No LGE	VT/VF arrest, secondary prevention ICD	IVCD	Negative	Negative, suspicious VUS in *TRPM4* c.2295dup
7	ACM/NDLVC	41–45	Non-dilated LV with hypokinesis Worst LVEF 15%, now 40% Ring-like intramural LGE	Non-sustained VT	Bifascicular block	Cardiomyopathy	Negative, VUS × 2
8	ACM/NDLVC	51–55	Non-dilated LV with hypokinesis LVEF now 46% Unable to assess LGE	PVCs>2500/day, Non-sustained VT, primary prevention ICD for syncope	Normal	Familial AF, cardiomyopathy, SCD	Negative, no rare variants
9	ACM/NDLVC	46–50	Non-dilated LV with hypokinesis LVEF 35% No LGE. Elevated T2	PVCs>9000/day Non-sustained VT	Normal	Familial AF	Negative, VUS × 2
10	DCM	31–35	Dilated LV with hypokinesis Worst LVEF 42%, now 48% No LGE	Negligible	Normal	SCD	Negative, VUS × 1
11	DCM	16–20	Dilated LV with hypokinesis Worst LVEF 42%, now 50% No LGE	PVCs > 1000/day, ventricular couplets	Normal	Negative	Negative, suspicious VUS in *TTN* c.58870G>A
12	DCM	31–35	Dilated LV with hypokinesis Worst LVEF 21%, now 60% No LGE, but elevated T1/T2	Negligible	Normal	Familial AF	Negative, VUS × 1
13	DCM	36–40	Non-dilated LV with hypokinesis Worst LVEF 28%, now 55% No LGE	Negligible	Normal	Familial AF	Negative, VUS × 4
14	DCM	56–60	Dilated LV with hypokinesis Worst LVEF 20%, now 50% Septal intramural LGE	Negligible	Normal	Familial AF, cardiomyopathy with heart transplant	Negative, no rare variants
15	DCM	36–40	Dilated LV/RV with hypokinesis Worst LVEF 10%, now 65% No LGE	Negligible	Normal	Negative	Negative, suspicious VUS in *SCN5A* c.5701 G>A
16	DCM	46–50	Dilated LV with hypokinesis Worst LVEF 10%, now 60% Unable to obtain cMRI	Negligible	Normal	Negative	Negative, VUS × 2
17	DCM	26–30	Non-dilated LV with hypokinesis Worst LVEF 35%, now 45% Unable to obtain cMRI	PVCs > 3000/day, ventricular couplets	Normal	Negative	Negative, heterozygous carrier status
18	DCM	36–40	Dilated LV with hypokinesis Worst LVEF 15%, now 50% No LGE	Negligible	Normal	Familial AF	Negative, Heterozygous carrier status
19	HCM	26–30	Asymmetric LVH LV septum 1.5 cm, posterior wall 1.2 cm No LGE, elevated T2	Negligible	Normal	Negative	Negative, heterozygous carrier status
20	HCM	41–45	Asymmetric LVH LV septum 1.5 cm, posterior wall 1.1 cm Septal intramural LGE, elevated T2	Negligible	Normal	HCM SCD	Negative, no rare variants
21	Idiopathic VF	26–30	Normal biventricular size and function Myxomatous mitral valve with bileaflet prolapse and trace MR	VT/VF arrest, Secondary prevention ICD	Normal	Negative	Negative, VUS × 1
22	PCCD	41–45	Normal biventricular size and function	Negligible	CHB requiring PPM	Negative	Negative, VUS × 1

All were diagnosed with AF prior to the development of a cardiomyopathy or channelopathy AF overlap syndrome.

All participants underwent a 12-lead ECG, 209 (85%) ambulatory monitoring, and 230 (93%) cardiac imaging according to the framework in *Figure 1*. Additional testing was performed in 112 participants (46%) with exercise treadmill ECG and 6 (2%) with a sodium channel blocker (procainamide) challenge. Findings from phenotypic evaluation including ECG, ambulatory ECG monitoring, and cardiac imaging are shown in *Table 5* and Supplementary data online, *Table S5* .

**Table 5:** Electrocardiogram, cardiac imaging, and ambulatory monitoring data

	Overall cohort	Genetic evaluation groups		
		Positive genetic testing	Gene elusive AF overlap syndrome	Negative genetic evaluation
Electrocardiogram	(*N* = 242)	(*N* = 48)	(*N* = 22)	(*N* = 172)
12-lead ECG interpreted as normal	128 (53%)	19 (40%)	8 (36%)	101 (59%)
Ventricular rate (b.p.m.)	69 (12)	69 (12)	71 (11)	68 (12)
PR interval (ms)	164 [151, 182]	164 [153, 188]	165 [144, 184]	163 [151, 179]
QRS duration (ms)	96 [89, 105]	97 [87, 110]	100 [93, 107]	95 [89, 104]
QRS morphology				
Normal	190 (79%)	29 (60%)	16 (73%)	145 (84%)
Fascicular block	19 (8%)	5 (10%)	2 (9%)	12 (7%)
Bundle branch block	29 (12%)	11 (23%)	3 (14%)	15 (9%)
Ventricular paced	4 (2%)	3 (6%)	1 (5%)	0 (0%)
QTc (ms)	424 [402, 446]	430 [405, 466]	430 [418, 456]	422 [401, 440]
QTc category^a^				
<470	217 (90%)	36 (75%)	18 (82%)	163 (95%)
470–499	13 (5%)	3 (6%)	4 (18%)	6 (4%)
≥500	4 (2%)	2 (4%)	0 (0%)	2 (1%)
Brugada pattern				
None	239 (99%)	47 (98%)	21 (96%)	171 (99%)
Non-diagnostic (types II or III) pattern	3 (1%)	1 (2%)	1 (5%)	1 (0.6%)
Diagnostic (type I) pattern	0 (0%)	0 (0%)	0 (0%)	0 (0%)
Low voltage QRS^b^	40 (17%)	11 (23%)	3 (14%)	26 (15%)
Left ventricular hypertrophy by QRS voltage	7 (3%)	1 (2%)	2 (9%)	4 (2%)
Ambulatory monitoring	(*N* = 209)	(*N* = 44)	(*N* = 16)	(*N* = 149)
Ventricular ectopic beats per day	21 [0, 205]	88 [6404]	65 [8, 1458]	12 [2132]
Ventricular ectopic beats per day				
<100	134 (64%)	23 (52%)	9 (41%)	102 (69%)
100–499	32 (15%)	9 (21%)	1 (5%)	22 (15%)
≥500	31 (15%)	10 (23%)	5 (31%)	16 (11%)
Complexity of ventricular ectopy				
None	43 (21%)	5 (8%)	2 (13%)	36 (24%)
Single PVCs only	87 (42%)	17 (33%)	7 (44%)	63 (42%)
Complex ectopy (couplets or NSVT)	71 (34%)	19 (43%)	7 (44%)	45 (20%)
Cardiac imaging	(*N* = 226)	(*N* = 46)	(*N* = 21)	(*N* = 159)
LV hypokinesis (LVEF < 50%)	34 (15%)	7 (15%)	15 (71%)	12 (8%)
LV dilation	14 (6%)	3 (7%)	7 (33%)	4 (3%)
Left ventricular hypertrophy				
Concentric LVH	25 (11%)	2 (4%)	4 (19%)	19 (12%)
Asymmetric septal hypertrophy	9 (4%)	2 (4%)	4 (19%)	3 (2%)
RV hypokinesis	20 (9%)	2 (4%)	10 (48%)	8 (5%)
RV dilation	16 (7%)	2 (4%)	3 (14%)	11 (7%)
LA enlargement	58 (26%)	13 (28%)	9 (43%)	36 (23%)
Significant valvular dysfunction^c^	3 (1%)	1 (2%)	1 (5%)	1 (0.6%)
Late gadolinium enhancement present^d^	35 (15%)	12 (29%)	5 (28%)	18 (16%)
Cardiac MRI parametric mapping of LV^d^				
Normal	93 (41%)	21 (50%)	3 (17%)	69 (60%)
Abnormal T1 time (normal: 940–1030 ms)	31 (14%)	12 (29%)	6 (34%)	13 (11%)
Abnormal T2 time (normal: 40–50 ms)	9 (4%)	1 (2%)	3 (17%)	5 (4%)

Continuous variables are listed as median [IQR] or mean (SD) and categorical variables are listed as number (percentage). Data include only probands who underwent genetic testing with a full cardiomyopathy and arrhythmia panel (*N* = 242; *Figure 1*).

^a^Excludes patients with QRS duration > 120.

^b^Defined as peak-to-peak QRS amplitude < 5 mm in II, III, and aVF or <10 mm in all precordial leads (V1–V6).

^c^Defined as severe stenosis or regurgitation of the aortic, tricuspid, or pulmonic valves; severe mitral regurgitation or mild or greater mitral stenosis.

^d^CMR available on 175 total patients (42 with positive genetic testing, 18 with gene elusive overlap syndromes, 115 negative genetic testing).

### Predictors of positive genetic testing during initial evaluation

**Figure 6:**
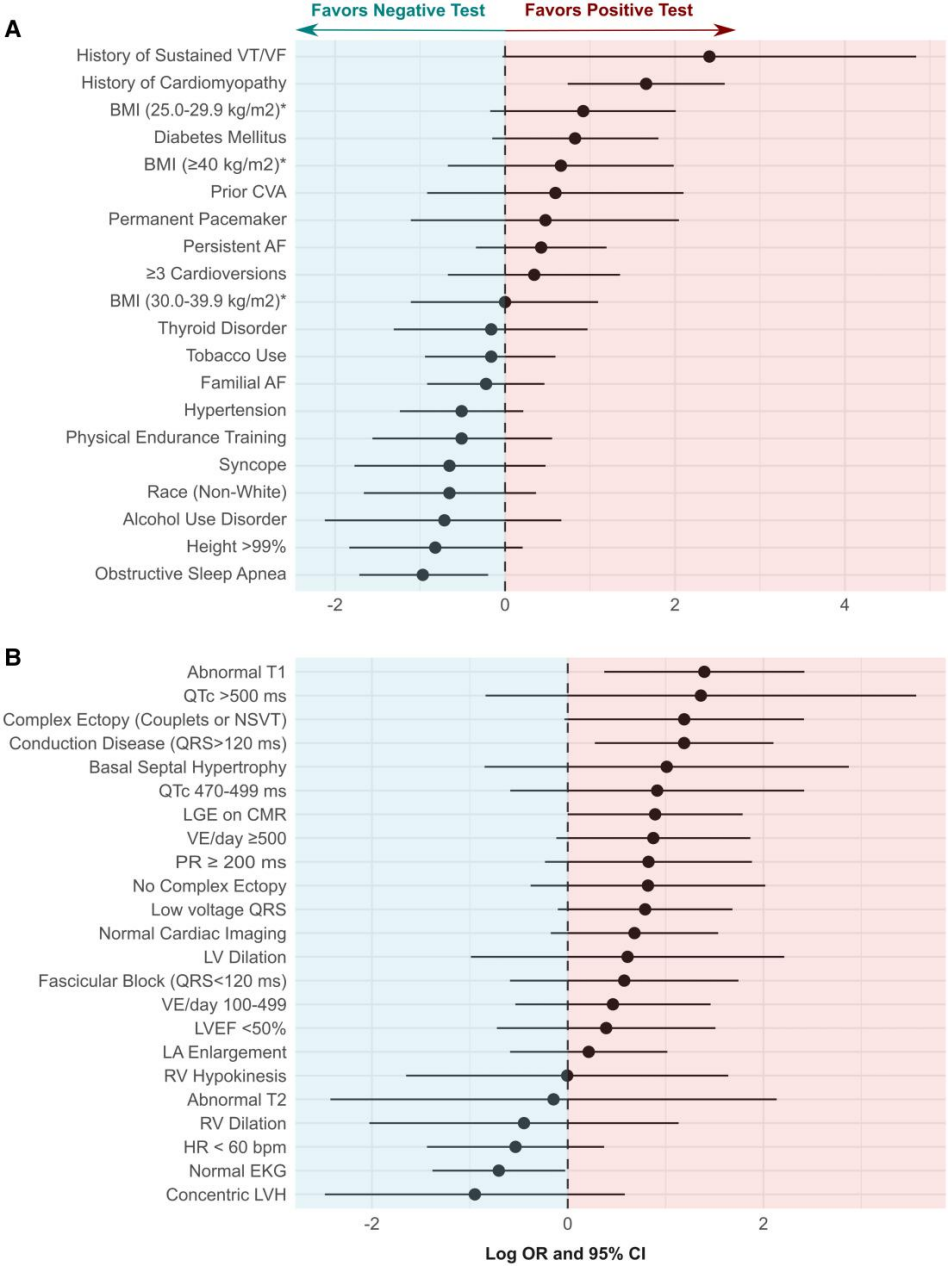
Forest plot of clinical and historical data (*A*) and electrocardiogram, cardiac imaging, ambulatory monitoring data (*B*) and the association with positive genetic testing. The analysis is adjusted for age at atrial fibrillation diagnosis, sex, and time between atrial fibrillation diagnosis and clinical genetic testing. Patients with gene elusive atrial fibrillation overlap syndromes were excluded from the prediction models. *Compared with normal range body mass index (20–25 kg/m^2^)

Multivariable logistic regression models were used to estimate the association between specific demographics and clinical characteristics and the likelihood of having positive genetic testing, adjusting for age at AF diagnosis, sex, and time between AF onset and clinical genetic testing (*Figure 6A*; Supplementary data online, *Table S7*). From the clinical history, a previous diagnosis of cardiomyopathy (OR 5.3, 95% CI 2.1–13.3, *P* < .001) was significantly associated with positive genetic testing. Family history of AF, type of AF (paroxysmal, persistent, permanent), and number of cardioversions were not significantly associated with positive genetic testing. Obstructive sleep apnoea (OSA) was the only clinical risk factor for AF that predicted negative genetic testing (OR 0.4, 95% CI 0.2–0.8, *P* = .013). The likelihood of a positive genetic test tended to be lower for patients with other clinical risk factors such as hypertension, height > 99th percentile, and alcohol use disorder that did not meet threshold for statistical significance. Tobacco use (OR 0.9, 95% CI 0.4–1.8) and obesity (BMI 30–39 kg/m^2^, OR 1.0, 95% CI 0.3–3.0; BMI > 40 kg/m^2^, OR 1.9, 95% CI 0.5–7.3) were found to have no significant associations with positive genetic testing.

### Predictors of positive genetic testing from electrocardiographic and imaging evaluation

Results from clinical phenotyping were also assessed in multivariable models adjusted for age at AF diagnosis, sex, and time from AF diagnosis to clinical genetic testing (*Figure 6B*). Findings from cardiac MRI were strongly associated with positive genetic testing, as the strongest predictor was elevated T1 time (OR 4.0, 95% CI 1.5–11.2, *P* = .007). Late gadolinium enhancement (LGE) from cardiac MRI was also associated with a positive genetic test (OR 2.4, 95% CI 1.0–6.0, *P* = .050). From the initial ECG, infranodal conduction disease (right bundle branch block/left bundle branch block/intraventricular conduction delay) was a significant predictor of positive genetic testing (OR 3.3, 95% CI 1.3–8.2, *P* = .011), while a 12-lead ECG at time of presentation interpreted as normal was associated with significantly lower likelihood of a positive genetic evaluation (OR 0.5, 95% CI 0.3–1.0, *P* = .042). Complex and frequent ventricular ectopy recorded on ambulatory monitoring was associated with increased likelihood of positive genetic testing, though this result did not reach statistical significance (OR 3.3, 95% CI 0.9–11.2, *P* = .057).

### Ventricular phenotype in participants with positive genetic testing

**Figure 7:**
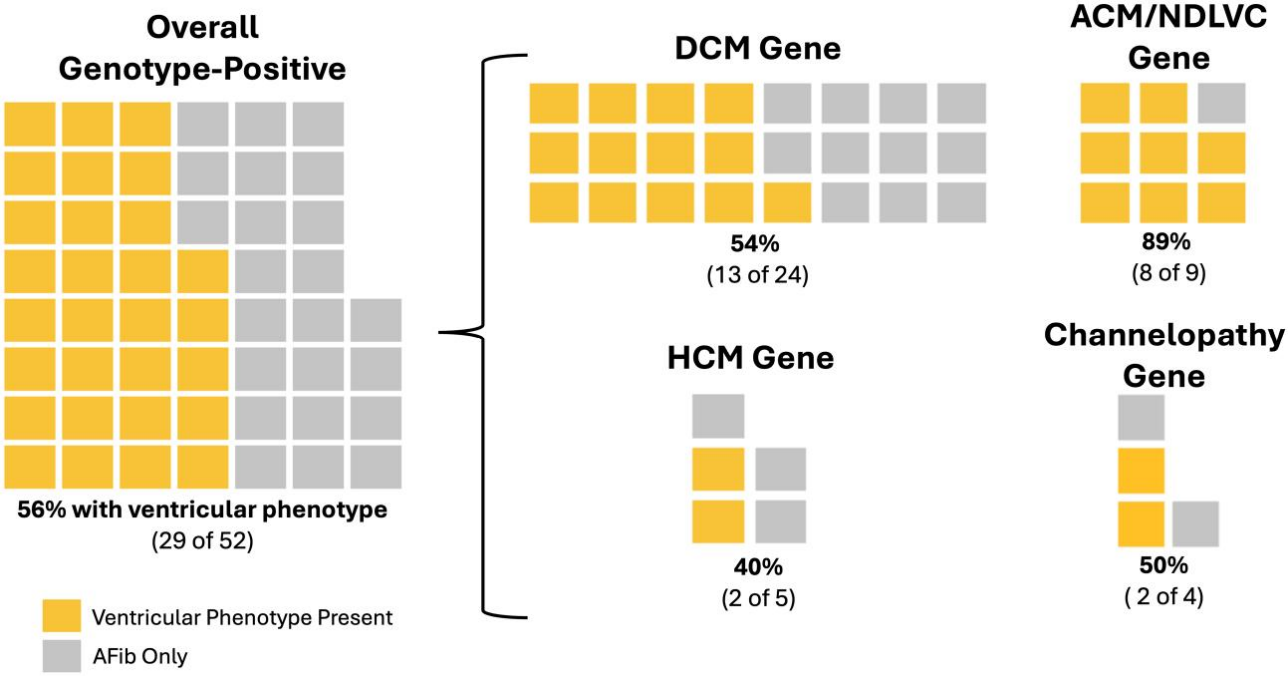
Penetrance of the ventricular phenotype. Each box represents one patient with positive genetic testing, including both probands (*N* = 48) and family members identified through cascade screening (*N* = 4). The penetrance of the ventricular phenotype was 56% in the overall cohort. When stratified according to specific atrial fibrillation genetic subgroups, penetrance of the ventricular phenotype ranged from 40% to 89%

After completion of genetic testing and phenotypic evaluation in probands and gene-positive family members (*N* = 246), penetrance of the ventricular phenotype in genotype-positive participants was assessed according to the predominant phenotype association shown in *Table 1*. Participants harbouring a variant in genes associated with ACM had the highest rate of ventricular involvement with 89% (8/9) found to have ACM as shown in *Figure 7* and Supplementary data online, *Figure S4*. While DCM-associated variants (including all P/LP *TTN* variants) were the most common result of positive genetic testing (all *TTN* in this cohort), 54% (13/24) of these participants had a DCM phenotype. Genes associated with HCM (*N* = 5) and channelopathies (*N* = 4) were identified less often but had similar rates of ventricular involvement with 40% of P/LP HCM variant carriers with overt phenotypic expression, and 50% of P/LP channelopathy participants found to have LQTS or BrS.

### Changes to clinical management

**Figure 8:**
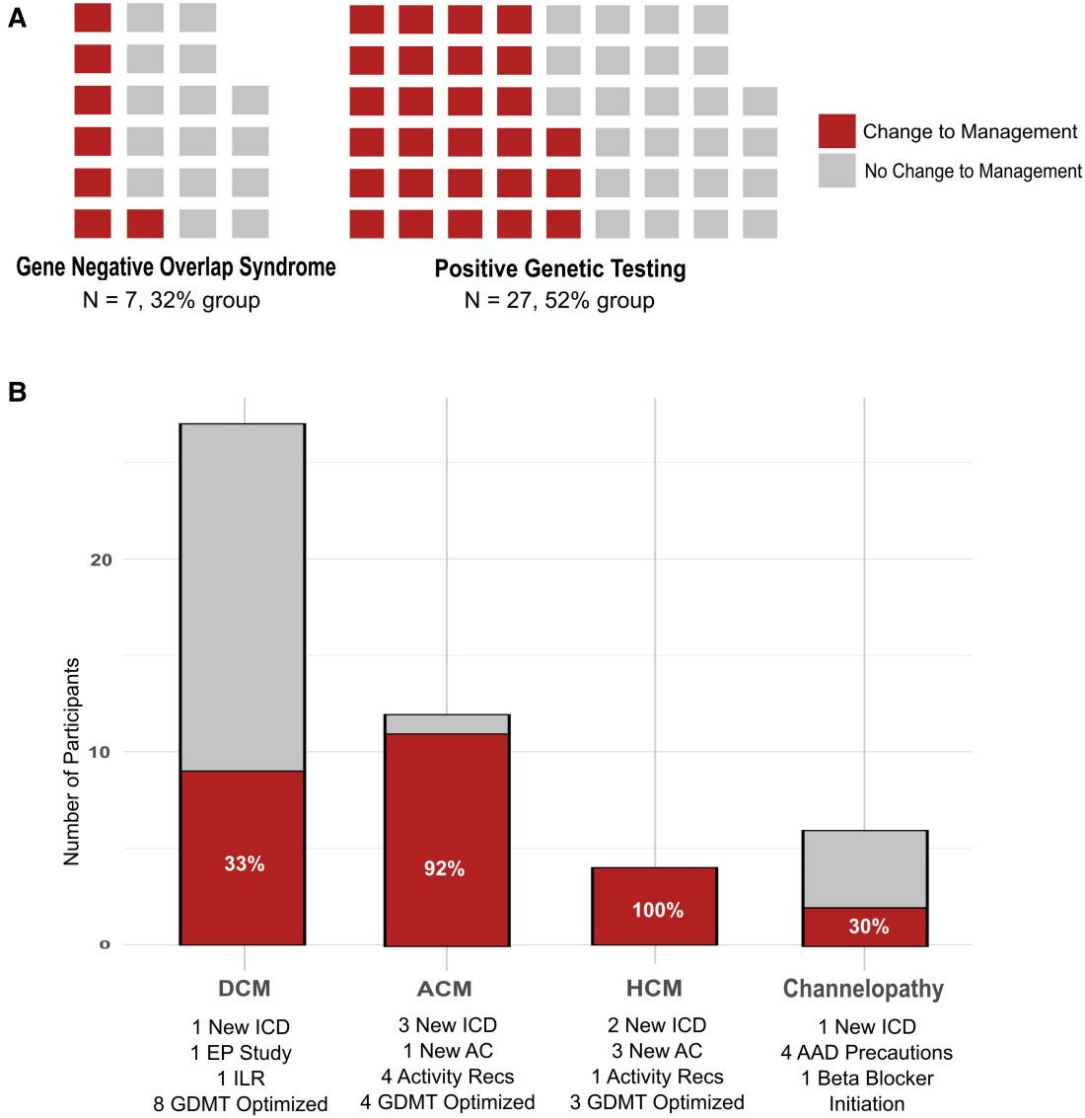
Management changes resulting from atrial fibrillation precision medicine clinic evaluation. Panel (*A*) shows the proportion of participants with a change in management stratified by gene elusive atrial fibrillation overlap syndrome (left figure) and those with positive genetic testing (right figure). Panel (*B*) shows the proportion of participants who had ≥1 change in their clinical management and summarizes the type of changes below

Impact on clinical care was assessed prospectively based on interventions performed after completion of the genetic and phenotypic evaluation. Complete details on changes to clinical management are presented in Supplementary data online, *Table S6*. Probands and family members with positive genetic testing had a change to management in 52% (27/52) of cases, and management was impacted in 11% (7/22) of participants with gene elusive AF overlap syndromes (*Figure 8A*). The frequency and type of clinical management were also different among the AF genetic subgroups with a phenotype present (*Figure 8B*). Ninety-two percent of participants with an ACM phenotype had a change to clinical management, most commonly initiation of guideline-directed medical therapy (GDMT) followed by recommendations for directed lifestyle intervention, such as exercise precautions for participants with desmosomal variants. Changes to clinical management were made less often (9 of 27, 33%) in participants with an identified DCM phenotype. Notably, all participants with HCM variants or phenotype had a change to clinical management. Results of the genetic evaluation facilitated early intervention to mitigate the risk of sudden cardiac death, with implantation of a new ICD in seven participants. For participants with channelopathy variants or phenotype, directed lifestyle interventions regarding medication use and precautions were the most common management change. Specifically, one participant had a pathogenic *KCNQ1* variant and was given precautions against QT-prolonging medications and three participants had a pathogenic *SCN5A* variant and were given precautions against sodium channel blocking medications and fever precautions.

## Discussion

We present here our approach to clinical and genetic testing, interpretation of results, and clinical decision-making in a dedicated AF precision medicine clinic. The 20% yield of positive genetic testing using a comprehensive cardiomyopathy/arrhythmia gene panel was twice the rate previously reported in unselected retrospective AF cohorts.^10,25,26^ The strongest predictors of positive genetic testing were history of cardiomyopathy, LGE or prolonged T1 time on cardiac MRI, and infranodal conduction disease seen on ECG, whereas the strongest predictors of negative genetic testing were history of OSA and a normal 12-lead ECG. Patients with positive genetic testing are susceptible to a genetic overlap syndrome; however, only a proportion will develop it. Here, we found the presence of a ventricular phenotype varied according to the AF genetic subgroup, and for most, it was ∼40%–50% (DCM genes, HCM genes, and channelopathy genes) but was higher for ACM genes at ∼90%. The results of genetic testing changed clinical management for the majority of patients with positive genetic testing, highlighted by seven patients in whom a new ICD was placed and 18 that had optimization or initiation of GDMT (*Structured Graphical Abstract*).

### Yield of genetic testing

Prior studies of AF at a population level have shown the yield of pathogenic variants in early-onset AF increases with younger age of diagnosis, approaching 5% with AF onset < 45 years of age.^27^ In more selected cohorts at AF referral centres, positive genetic testing in patients with early-onset AF was ∼10% in a predominately White cohort and slightly less in an ethnically diverse cohort.^10,25^ The significantly higher rate of positive genetic testing in this prospective cohort (20%) may be accounted for by two key differences. First, younger age at AF diagnosis is associated with a higher likelihood of positive genetic testing and the median age of AF diagnosis for patients referred to our clinic was 39 years of age compared to 50 years of age for our prior retrospective study.^10^ Second, participants in this study were specifically referred to the AF Precision Medicine Clinic for genetic evaluation by clinicians after completing a baseline evaluation for AF, which may have identified clinical features that raised suspicion for a genetic aetiology. These results are consistent with another published cohort prospectively referred to a genetics clinic that found 24% had positive genetic testing.^26^ As healthcare systems and providers consider incorporating new recommendations for genetic testing in patients with early-onset AF,^11,12^ a yield of positive genetic testing of 20% for AF is comparable to that of other cardiac phenotypes for which genetic testing is already routinely used, such as DCM and BrS.^28,29^

### Gene elusive atrial fibrillation overlap syndromes

Patients diagnosed with an AF overlap syndrome but have negative genetic testing are termed gene elusive. The percentage of patients with gene elusive DCM is estimated to be ∼50%–80%, ACM is ∼40%–50%, HCM is ∼40%, LQTS is ∼15%–30%, and BrS is ∼80%. The rate of gene elusive syndromes is known to be even higher in patients from non-European genetic ancestry.^12,21–24^ In our study, 22 participants (9%) were found to have a gene elusive AF overlap syndrome, which included 5 (23%) from a non-European genetic ancestry.

Among the gene elusive participants, four (18%) had a suspicious VUS and seven (32%) had a positive family history suspicious for an inherited cardiomyopathy/arrhythmia syndrome. Taken together, 50% of the gene elusive participants had no suspicious genetic variant or family history to suggest an AF genetic overlap syndrome, supporting the added benefit of clinical testing with cardiac MRI and ambulatory monitoring rather than reserving additional testing only for those with positive genetic testing.

### Predictors of positive and negative genetic testing

Using clinical information available during the initial evaluation, the strongest predictors of positive genetic testing were personal history of ventricular arrhythmias (sustained ventricular tachycardia/fibrillation > non-sustained ventricular tachycardia or couplets) or ventricular cardiomyopathy. These suggest the presence of a pathogenic variant because they are manifestations of a ventricular phenotype of the cardiomyopathy genes sequenced in our panel.^14^ Notably, family history of AF was not a predictor of positive genetic testing, likely because AF has a complex genetic architecture with polygenic risk and shared clinical risk factors contributing more to heritability than rare, large-effect sized variants.^12^

Alternatively, strong clinical risk factors for AF are expected to lower the yield of genetic testing.^14^ Consistent with this idea, the strongest predictors of negative genetic testing were OSA, tall height (>99th percentile), and alcohol use disorder. However, although OSA was the strongest predictor of negative genetic testing, a relatively large proportion of them (14%) still carried a pathogenic variant. This suggests rare pathogenic variants confer genetic susceptibility that still requires a ‘second hit’ to develop AF.^12^ Prior work has demonstrated that AF polygenic risk can increase the risk for development of AF in rare variant carriers.^30^ However, risk factors for incident AF may also contribute to development of the clinical phenotype consistent with the findings from this cohort. In many cases, the ‘second hits’ are these common clinical risk factors and genetic testing should not be ruled out solely due to their presence. Clues to underlying genetic status were also identified through the detailed phenotype evaluation. Conduction disease on a 12-lead ECG manifesting as left bundle branch block, right bundle branch block, or intraventricular conduction delay was a significant predictor of positive genetic testing as many genes encode proteins responsible for electrical transduction. One of the strongest predictors of negative genetic testing was a normal 12-lead ECG. Careful interpretation should be used to not overlook subtle ECG abnormalities found in early-stage genetic cardiomyopathies such as abnormal QRS voltage (low or high) and fascicular blocks.^18^

Using information from cardiac imaging, identification of ventricular fibrosis from cardiac MRI with elevated T1 times or ventricular LGE was found to be one of the strongest predictors of positive genetic testing. Interestingly, the association for abnormal T1 appears stronger than LGE, consistent with research in non-ischaemic cardiomyopathy showing T1 mapping is superior to LGE for assessment of diffuse fibrosis.^31^ In this clinical population, 71% (34 out of 48) of participants with positive genetic testing harboured a cardiomyopathy variant that may benefit from early detection of adverse remodelling through T1 mapping rather than LGE alone. Although not significant, right ventricular dilation and concentric left ventricular hypertrophy were predictors of negative genetic testing. These imaging features are consistent with diastolic dysfunction and suggest adverse atrial remodelling that predisposes to development of AF and when coupled with shared metabolic risk factors may be more suggestive of negative genetic testing.

Taken together, our results suggest that individual risk factors are not strong enough predictors to determine whether or not a patient should undergo genetic testing. However, given the time between AF onset and evaluation, there may be unmeasured confounders further affecting the risk of phenotype development. Selective availability of genetic testing and variable timing from AF diagnosis to evaluation may also introduce selection and time-related biases not fully addressed by adjustment. Accordingly, these findings should be interpreted as associations. The associations that were identified suggest a multivariable model may be developed in the future to guide clinical decision-making.

### Penetrance of the ventricular phenotype for atrial fibrillation genetic overlap syndromes

Population-based studies have shown that rare cardiomyopathy-associated variants may be implicated in the pathogenesis of an atrial myopathy leading to AF, rather than causing AF through progressive left ventricular dysfunction.^32^ Identification of a ventricular phenotype in those with positive genetic testing is thus a critical part of the evaluation, as not all variant carriers will develop ventricular disease. For participants with positive genetic testing in this cohort, the median time from AF diagnosis to evaluation in clinic was ∼4 years, but the absolute range was 1 week up to 43 years. This variability may affect penetrance estimates because prior studies suggest the natural history of developing the ventricular phenotype for specific cardiomyopathy genes may significantly differ. For example, HCM may present earlier in genotype-positive patients (*MYH7*, *MYBPC3*) than DCM does in patients with pathogenic *TTN* variants.^33^ Conversely, P/LP variants associated with DCM may present later in life, as other studies have found pathogenic *TTN* variants contribute to a significant proportion of late-onset DCM (>60 years of age).^34,35^ We found the penetrance of a ventricular phenotype for probands with a pathogenic *TTN* variant and those identified through cascade screening was 54% (13 out of 24). However, the penetrance of the ACM-gene subgroup was higher at ∼90% (8 out of 9), consistent with other studies that have found the penetrance of ACM genes, predominantly *LMNA*, *FLNC*, and *DSP*, are higher than the penetrance of *TTN* for DCM.^34,36^

### Changes to clinical management for patients with positive genetic testing

Early-onset AF has been shown to associate with increased risk of development of cardiomyopathy, stroke, and heart failure.^37^ Identification of a pathogenic variant or gene elusive overlap syndrome allows an opportunity to intervene and potentially mitigate these adverse events. In this cohort, 52% of patients with positive genetic testing had a change in their clinical management.

One of the most consequential decisions in caring for patients with genetic cardiomyopathy and arrhythmia syndromes is whether to implant an ICD. In patients with early-onset AF who undergo genetic testing, positive results can introduce special indications for ICD and pacemaker placement. First, ICD placement is considered reasonable in genetic cardiomyopathies associated with *LMNA*, *FLNC*, *PLN*, and desmosomal genes if the left ventricular ejection fraction is <45% and other clinical risk factors are present.^38–41^ One patient in our study underwent new ICD implantation due to this indication. Second, for patients diagnosed with ACM who experience syncope associated with ventricular arrhythmias, an ICD is reasonable.^38–41^ One patient in our study underwent new ICD implantation due to this indication. Third, for patients diagnosed with HCM, special primary prevention ICD indications for HCM apply.^40–42^ Two patients in our study were given a new diagnosis of HCM and underwent new ICD implant after individualized risk stratification. Finally, for patients with an indication for permanent pacing who have a pathogenic variant associated with a high risk for ventricular arrhythmias, an ICD with pacing capabilities is reasonable.^20,38^ This indication applied to one patient in our study who underwent a new ICD implant and two patients who underwent an upgrade from a pacemaker to ICD. While indications for primary prevention ICD vary between the USA and Europe, both guidelines recommend using genotype and individualized risk factors to guide patient centred risk–benefit discussion that applied to all patients with primary prevention ICD in this study. Other special indications exist for other genetic syndromes but were not encountered for our current study.

For most patients with AF, anticoagulation for stroke prophylaxis is guided by the CHA_2_DS_2_-VA score or other similar risk estimators.^43^ However, special recommendations exist to anticoagulate patients with HCM or ACM regardless of baseline embolic stroke risk predicted by conventional risk calculators.^38,42^ In our study, seven participants with a CHA_2_DS_2_-VA score of ≤1 were given a new diagnoses of HCM or ACM and started on anticoagulation for stroke prophylaxis.

It is currently unknown how long to continue GDMT in patients who have depressed left ventricular function in the setting of AF that recovers with successful rhythm control therapy (e.g. arrhythmia-induced or tachycardia-induced cardiomyopathy). We have previously shown that for patients with AF and pathogenic *TTN* variants who developed any degree of left ventricular systolic dysfunction, 80% had symptomatic heart failure at the end of follow-up, among which approximately one third had NYHA class III/IV heart failure or had undergone cardiac transplant.^18^ Patients with *TTN* (+) DCM are known to respond well to GDMT; thus, our practice was to proactively optimize GDMT for patients with pathogenic *TTN* or other cardiomyopathy variants who had any history of left ventricular systolic dysfunction.^44^ Following shared decision-making, seven patients were started on beta-blockers and/or angiotensin-converting enzyme inhibitors/angiotensin receptor blockers to prevent recurrence or progression of left ventricular systolic dysfunction.

Those with a positive genetic evaluation can also benefit from directed lifestyle interventions that can reduce the risk of disease progression and mitigate development of ventricular arrhythmic events. In patients with genetic cardiomyopathy and arrhythmia syndromes, guidance regarding exercise restriction is becoming more lenient as emerging data support the safety of mild/moderate exercise for specific syndromes (e.g. non-obstructive HCM, LQTS).^45,46^ However, concern remains that intense, endurance exercise can promote development or progression of disease, and participation should only be considered in consultation with a provider specialized in these conditions.^47,48^ The strongest data support exercise restriction to ≤6 metabolic equivalents in patients with pathogenic desmosomal variants with ARVC, but these results have been generalized to also include left ventricular and biventricular forms of ACM.^38^ This affected four participants with ACM in our study.

Patients with LQTS or BrS, or pathogenic variants in genes associated with either syndrome, are recommended to avoid medications that prolong QT or block the cardiac potassium or sodium channels, respectively.^40,47^ We recommended avoidance of Class I antiarrhythmics (e.g. flecainide, propafenone), which block the cardiac sodium channel, in three patients with pathogenic *SCN5A* variants. Avoidance of Class III antiarrhythmics (e.g. sotalol, dofetilide), which block cardiac potassium channels, was also recommended in one participant with a pathogenic *KCNQ1* variant.

### Limitations

This was a single-centre experience reporting findings from a newly established AF Precision Medicine Clinic. Thus, these findings may vary between centres. Approximately 90% of the study participants were White/non-Hispanic, limiting the generalizability of these findings to more diverse populations. Small subgroup sizes limited power and reduced precision. Several predictors—including prior sustained ventricular arrhythmia—had wide confidence intervals despite effect estimates consistent with increased odds of a positive genetic test. Future studies with increased sample sizes are warranted to study predictors for patients who warrant referral to a specialty AF Precision Medicine Clinic. Finally, long-term follow-up is needed to analyse whether genetically informed medical interventions improved clinical outcomes.

## Conclusion

Here, we describe our current framework for evaluation of patients presenting with early-onset AF in a dedicated AF precision medicine clinic. We demonstrate that features from the patient’s history can raise suspicion for genetic disease and that the presence of clinical risk factors for AF does not exclude a genetic diagnosis. Genetic evaluation may have diagnostic and clinical management implications for patients with early-onset AF.

## Supplementary Material

ehaf829_Supplementary_Data

## Declarations

### Data Availability

The data underlying this article cannot be shared publicly for the privacy of patients that participated in the registry. The data will be shared on reasonable request to the corresponding author.

### Pre-registered Clinical Trial Number

NCT05190679OR.
