# Supplementary material for: Genetic evaluation of early-onset atrial fibrillation: impact on patient management
Source: Eur Heart J. 2025 Oct 30;47(13):1554–72. doi: 10.1093/eurheartj/ehaf829 (PMC12814854; doi:10.1093/eurheartj/ehaf829)
Supplement: ehaf829_Supplementary_Data [file ehaf829_supplementary_data.docx]

**Genetic evaluation of early-onset atrial fibrillation:**

**impact on patient management**

**SUPPLEMENTARY DATA**

Section S.1: Supplementary Methods

Supplemental Table 1: Complete list of genes grouped according to AF genetic subgroups

Supplemental Table 2: Diagnostic criteria and management considerations for Inherited Syndromes

Supplemental Figure 1: AFPMC Best Practice Alert through Electronic Medical Record

Supplemental Figure 2: Percentage of patients referred to the AF Precision Medicine Clinic by age

Supplemental Figure 3: Temporal Trends on number of AF Precision Medicine Clinic referrals

Supplemental Table 3: Details of genetic testing and full summary of results

Supplemental Table 4: Suspicious variants of undetermined significance (VUS)

Supplemental Table 5: Details of clinical testing

Supplemental Figure 4: Penetrance of the ventricular phenotype in probands

Supplemental Table 6: Details of phenotype penetrance and changes to management

Supplemental Table 7: Summary statistics of regression models for clinical predictors of positive genetic testing

Section S.2: Supplemental References

**Supplementary Methods S.1**

Referral to the AF Precision Medicine Clinic

Participants were referred for genetic evaluation of AF between October 2020 to September 2024. Referrals were accepted from treating cardiologists or by self-referral. The AF Precision Medicine Clinic, which was formally established in April 2023, sees more than 350 new patients per year. Referral to the clinic was placed by the patients’ cardiologists, often in response to a best practice alert (BPA). The BPA is an automated alert that appears in the electronic medical record for young patients with atrial fibrillation and incorporates eligibility criteria to select patients that we recommend be considered for genetic evaluation. (**Supplemental Figure 1**). **Supplemental Figure 2** demonstrates the referral percentage of eligible young patients with AF was similar across age groups. There was considerable initial uptake of referral through the BPA, which fell over time but has remained at a steady level as shown in **Supplemental Figure 3**. Patients in the clinic were prospectively enrolled in an IRB-approved registry (IRB #201666, NCT05190679). Participant data were recorded in a password-protected database designed for clinical research (REDCap).(1) Some family members underwent targeted genotyping limited to only the familial variant. Depending on the results of the standard clinical evaluation and genetic testing, additional diagnostic testing with an exercise tolerance test, extended ECG monitoring, or a procainamide challenge was performed. Patients were seen for a 3-month follow-up appointment either in-person or by telemedicine to review the results of their tests and develop a longitudinal care plan.

Genetic Sequencing and Variant Interpretation

Most patients underwent clinical genetic testing with a comprehensive cardiomyopathy and arrhythmia panel using commercial vendors (Labcorp/Invitae, Burlington, NC; GeneDx, Stamford, CT) or the CLIA-approved Vanderbilt University Medical Center Clinical Genetic Testing Laboratory (Nashville, TN). Few patients (N = 6) had smaller dedicated cardiac panels sent prior to comprehensive evaluation in the AF Precision Medicine Clinic. The genes included in the genetic testing panel differed by commercial genetic testing laboratory: GeneDx = 124 genes, Invitae/Labcorp = 157 genes and VUMC Clinical Genetic Testing Laboratory = 175 genes. All panels included genes with definitive/strong level of evidence shown in Table 1 with variable inclusion of disputed and preliminary evidence genes shown in Supplemental Table 1. The genes included in the respective panels did not change over the course of the study. All genetic platforms utilized for the study used next-generation sequencing (NGS) and additional confirmatory testing dependent on variant calls. While there is vendor variability in sequencing methodology, all platforms required read depth of at least 50x and intronic coverage of at least 10 base pairs either side of coding exons in addition to rigorous quality control metrics. Variants are filtered using well-validated but proprietary technology specific to each vendor (2–4). Filtered variants are then reviewed by genetic scientists and clinicians before variants are returned to the clinical team in the final result.

The clinical genetic testing laboratories and investigators used the ACMG/AMP (American College of Medical Genetics and Association for Molecular Pathology) Standards and Guidelines for variant interpretation.(5) The ACMG/AMP criteria designate a variant as pathogenic (P), likely-pathogenic (LP), a variant of undetermined significance (VUS), likely-benign (LB), or benign (B). If any variants were officially reclassified by the clinical genetic testing laboratory, the current classification as of 2/20/2025 was used for this analysis.

“Positive” genetic testing was defined by any of the following conditions:

- One or more P/LP variants in a gene with autosomal dominant (AD) inheritance, or
- Two P/LP variants in a gene with autosomal recessive (AR) inheritance, or
- One P/LP variant in hemizygous men or homozygous women for genes with X-linked inheritance.

AF Genetic Subgroups

Genes were classified according to the predominant gene-phenotype association here termed “AF genetic subgroups”. This is a challenging clinical issue, as there is often overlapping genetic architecture of distinct phenotypes (eg ACM/NDLVC genes may have LV dilation as with DCM), and also different variants within the same gene can cause different phenotypes (eg *MYH7* is both a definitive evidence gene for DCM and HCM.) Categorized of “AF genetic subgroups” was done by gene-level predominant phenotypic association to facilitate patient management according to genetic susceptibility for syndromes with established guidelines that inform clinical management. Genes associated with syndromes that are less common in our study population and genes with only limited, preliminary, refuted, or disputed evidence for their gene-disease association according to ClinGen were not assigned to an AF genetic subgroup.

**SUPPLEMENTAL TABLE 1: Complete list of genes (N=175) for comprehensive cardiac genetic testing panels.** Individual genes tested varied by vendor. This table lists the most comprehensive gene panel utilized by the Vanderbilt Clinical Genetics Laboratory.

| *A2ML1*  *ABCC9*  *ACADVL*  *ACTC1*  *ACTN2*  *AGL*  *AKAP9*  *ALMS1*  *ALPK3*  *ANK2*  *ANKRD1*  *BAG3*  *BRAF*  *CACNA1C*  *CACNA2D1*  *CACNB2*  *CALM1*  *CALM2*  *CALM3*  *CALR3*  *CASQ2*  *CAV3*  *CAVIN4*  *CBL*  *CHRM2*  *CPT2*  *CRYAB*  *CSRP3*  *CTF1*  *CTNNA3*  *DEPDC5*  *DES*  *DMD*  *DNAJC19*  *DOLK* | *DSC2*  *DSG2*  *DSP*  *DTNA*  *ELAC2*  *EMD*  *EYA4*  *FHL1*  *FHL2*  *FKRP*  *FKTN*  *FLNC*  *GAA*  *GATA4*  *GATA5*  *GATA6*  *GATAD1*  *GJA5*  *GLA*  *GNB5*  *GPD1L*  *HCN4*  *HFE*  *HRAS*  *ILK*  *JPH2*  *JUP*  *KCNA1*  *KCNA5*  *KCND3*  *KCNE1*  *KCNE2*  *KCNE3*  *KCNE5*  *KCNH2* | *KCNJ2*  *KCNJ5*  *KCNJ8*  *KCNK3*  *KCNQ1*  *KCNQ2*  *KCNQ3*  *KCNT1*  *KRAS*  *LAMA4*  *LAMP2*  *LDB3*  *LMNA*  *LRRC10*  *MAP2K1*  *MAP2K2*  *MED12*  *MIB1*  *MTND1*  *MTND5*  *MTND6*  *MTO1*  *MTTD*  *MTTG*  *MTTH*  *MTTI*  *MTTK*  *MTTL1*  *MTTL2*  *MTTM*  *MTTQ*  *MTTS1*  *MTTS2*  *MYBPC3*  *MYH6* | *MYH7*  *MYL2*  *MYL3*  *MYL4*  *MYLK2*  *MYOM1*  *MYOZ2*  *MYPN*  *NEBL*  *NEXN*  *NF1*  *NKX2-5*  *NPPA*  *NRAS*  *PCDH19*  *PDLIM3*  *PKP2*  *PLEKHM2*  *PLN*  *PPA2*  *PRDM16*  *PRKAG2*  *PRRT2*  *PTPN11*  *RAF1*  *RANGRF*  *RASA1*  *RBM20*  *RIT1*  *RRAS*  *RYR2*  *SCN10A*  *SCN1A*  *SCN1B*  *SCN2B* | *SCN3B*  *SCN4B*  *SCN5A*  *SCN8A*  *SCN9A*  *SDHA*  *SGCD*  *SHOC2*  *SLC22A5*  *SLC2A1*  *SLMAP*  *SNTA1*  *SOS1*  *SOS2*  *SPRED1*  *TAZ*  *TBX20*  *TBX5*  *TCAP*  *TECRL*  *TGFB3*  *TMEM43*  *TMEM70*  *TMPO*  *TNNC1*  *TNNI3*  *TNNT2*  *TOR1AIP1*  *TPM1*  *TRDN*  *TRPM4*  *TTN*  *TTR*  *TXNRD2*  *VCL* |
| --- | --- | --- | --- | --- |

**SUPPLEMENTAL TABLE 2: Diagnostic criteria and management considerations used for inherited syndromes** are based on international guidelines and task force recommendations. Note, Arrhythmogenic Cardiomyopathy (ACM) is used to describe both Non-Dilated LV Cardiomyopathy (NDLVC) and Arrhythmogenic Right Ventricular Cardiomyopathy (ARVC) phenotypes.

| ***Syndrome*** | ***Diagnosis*** | ***Management Considerations*** | ***Citations*** |
| --- | --- | --- | --- |
| **Dilated Cardiomyopathy (DCM)** | LV dilation with impaired LV function (LVEF <50%) | HFrEF GDMT  SCD Risk Stratification  Stroke Risk Reduction | 2023 ESC (6)  2022 AHA/ACC/HFSA (7) |
| **Non-dilated Left Ventricular Cardiomyopathy**  **(NDLVC)** | Impaired LV systolic function without LV dilation, or LV fibrosis by DE-CMR | HFrEF GDMT  SCD Risk Stratification  Stroke Risk Reduction | 2023 ESC (6)  2022 AHA/ACC/HFSA (7) |
| **Hypertrophic Cardiomyopathy**  **(HCM)** | Increased LV wall thickness (>1.5 cm men, >1.3 cm women, >1.3 cm relative of genotype positive family member) | SCD Risk Stratification  Stroke Risk Reduction  LVOT Obstruction | 2024 AHA/ACC/  AMSSM/HRS/PACES/  SCMR (8) |
| **Arrhythmogenic Right Ventricular Cardiomyopathy (ARVC)** | *Definite:* 2 major, or 2 major + 1 minor, or 4 minor  *Borderline:* 1 major + 1 minor, or 3 minor  *Possible:* 1 major, or 2 minor | SCD Risk Stratification  Exercise Restriction  Stroke Risk Reduction | 2010 Modified Taskforce Criteria (9)  2023 ESC (6) |
| **Arrhythmogenic Cardiomyopathy**  **(ACM)** | Cardiomyopathy with arrhythmia predominant phenotype. Used in this manuscript to encompass both NDLVC and ARVC. | Per NDLVC and ARVC phenotype | 2019 HRS (10) |
| **Brugada Syndrome** | Type I EKG pattern spontaneously or with sodium channel blocker challenge | SCD Risk Stratification  Drug Precautions | 2013 HRS/EHRA/APHRS (11) |
| **Long QT Syndrome** | 1. LQTS risk score >3.5 *or*  2. Pathogenic LQTS variant *or*  3. QTc >500 ms without secondary cause | SCD Risk Stratification  Drug Precautions | 2013 HRS/EHRA/APHRS (11) |
| **Catecholaminergic Polymorphic Ventricular Tachycardia (CPVT)** | 1. Exercise-induced bidirectional VT or PMVT *or*  2. Pathogenic CPVT variant with exercise induced PVCs or VT | Exercise Restriction  SCD Risk Stratification  AAD Therapies | 2013 HRS/EHRA/APHRS  (11) |
| **Progressive Cardiac Conduction Disease (PCCD)** | Unexplained progressive conduction abnormalities age <50y in the absence of skeletal myopathies | SCD Risk Stratification | 2013 HRS/EHRA/APHRS  (11) |
| **Hemochromatosis** | Serum ferritin >200 ng/mL (women) or >300 (men), *and* Tsat > 45%, *and* two pathogenic HFE variants | Therapeutic phlebotomy | 2019 ACG (12)  2018 HFE International Taskforce (13) |
| **Amyloidosis** | Histologic evidence of TTR amyloid deposition, or imaging consistent with cardiac amyloid deposition and pathogenic TTR variant | TTR-directed therapy | 2023 ACC Expert Consensus (14) |
| HFrEF= heart failure with reduced ejection fraction. GDMT=guideline directed medical therapy. SCD=sudden cardiac death. ESC=European Society of Cardiology. AHA=American Heart Association. ACC=American College of Cardiology. HFSA=Heart Failure Society of America. DE-CMR= delayed enhancement cardiac MRI. LVOT=LV outflow tract. AMSSM= American Medical Society for Sports Medicine. HRS=Heart Rhythm Society. PACES= Pediatric and Congenital Electrophysiology Society. SCMR= Society for Cardiovascular Magnetic Resonance. EHRA=European Heart Rhythm Association. APHRS=Asian Pacific Heart Rhythm Society. ACG=American College of Gastroenterology. AAD = antiarrhythmic drug. TSAT= transferrin saturation. *HFE*=homeostatic iron regulator gene. *TTR*= transthyretin gene. | | | |

**SUPPLEMENTAL FIGURE 1:** Best practice alert example for a 37-year-old patient without other clear etiologies identified from automated ICD/CPT code review for referral to the Atrial Fibrillation Precision Medicine Clinic.

 
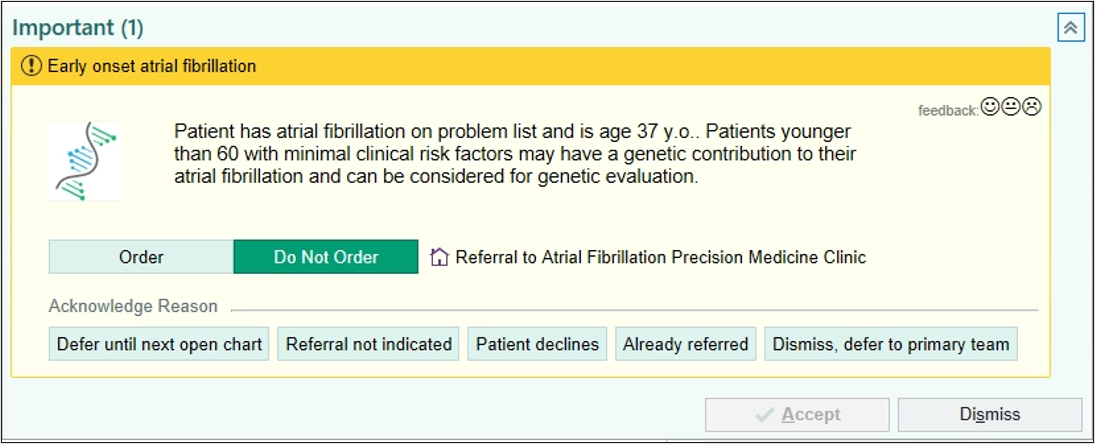


**Supplemental Figure 2**: Percentage of patients referred to the AF Precision Medicine Clinic by age

**
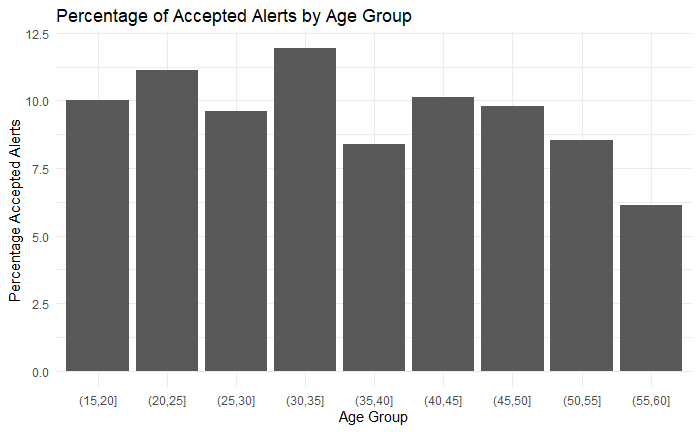
**

**Supplemental Figure 3**: Temporal Trends on number of AF Precision Medicine Clinic referrals

**
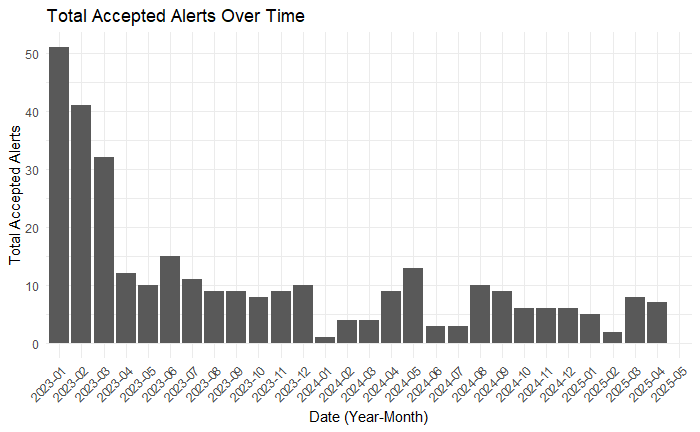
**

**SUPPLEMENTAL TABLE 3: Details of genetic testing and full summary of results**

|  | **Total**  **Cohort**  **(N=246*)** | **Genetic Evaluation Groups** | | |
| --- | --- | --- | --- | --- |
|  |  | **Positive**  **Genetic Testing**  **(N=52)** | **Gene Elusive AF Overlap**  **Syndrome**  **(N=22)** | **Negative**  **Genetic Evaluation**  **(N=172)** |
| Type of testing  Multi-gene CM/arrhythmia panel  Targeted genotyping | 242 (98%)  4 (2%) | 48 (92%)  4 (8%) | 22 (100%)  0 (0%) | 172 (100%)  0 (0%) |
| Genetic testing laboratory  VUMC Clinical Genetics Lab  Invitae  GeneDx  Other | 60 (24%)  115 (47%)  69 (28%)  2 (0.8%) | 12 (23%)  12 (23%)  16 (31%)  1 (1.9%) | 8 (36%)  8 (36%)  6 (27%)  1 (4.5%) | 40 (23%)  40 (23%)  47 (27%)  0 (0%) |
| Number of genes on panel | 157 [76,197] | 157 [82,175] | 157 [76,175] | 157 [100,197] |
| Number of variants reported | 1 [0,6] | 2.50 [1,5] | 1 [0,4] | 1 [0,6] |
| Genetic testing results  Positive  Negative, with a VUS reported  Negative, and carrier for an AR syndrome  Negative, with no rare variants reported | 52 (21%)  129 (52%)  18 (7%)  47 (19%) | 52 (100%)  0 (0%)  0 (0%)  0 (0%) | 0 (0%)  15 (68%)  3 (14%)  4 (18%) | 0 (0%)  114 (66%)  15 (9%)  43 (25%) |
| Compound or digenic heterozygote | 1 (0.4%) | 1 (1.9%) | 0 (0%) | 0 (0%) |
| VUS reported | 162 (66%) | 33 (64%) | 15 (68%) | 114 (66%) |
| “Suspicious” VUS | 36 (15%) | 6 (12%) | 4 (18%) | 26 (15%) |
| Heterozygous carrier of pathogenic AR gene | 60 (24%) | 13 (25.0%) | 7 (32%) | 40 (23%) |
| Tested for Myotonic Dystrophy | 31 (13%) | 6 (12%) | 3 (14%) | 22 (13%) |
| Gene for heterozygous AR carriers  *FKRP*  *GAA*  *HFE*  *SGCD*  *TRDN*  *ACADVL*  *ALMS1*  *DNAJC19*  *ELAC2*  *MYPN*  *PPA2*  *SLC22A5* | 2 (0.8%)  6 (2.4%)  42 (17%)  1 (0.4%)  1 (0.4%)  2 (0.8%)  1 (0.4%)  1 (0.4%)  1 (0.4%)  1 (0.4%)  1 (0.4%)  1 (0.4%) | 1 (1.9%)  1 (1.9%)  9 (17%)  1 (1.9%)  1 (1.9%)  0 (0%)  0 (0%)  0 (0%)  0 (0%)  0 (0%)  0 (0%)  0 (0%) | 0 (0%)  0 (0%)  7 (32%)  0 (0%)  0 (0%)  0 (0%)  0 (0%)  0 (0%)  0 (0%)  0 (0%)  0 (0%)  0 (0%) | 1 (0.6%)  5 (2.9%)  26 (15%)  0 (0%)  0 (0%)  2 (1.2%)  1 (0.6%)  1 (0.6%)  1 (0.6%)  1 (0.6%)  1 (0.6%)  1 (0.6%) |

**SUPPLEMENTAL TABLE 4: Suspicious variants of undetermined significance (VUS)**

| **Patient** | **Suspicious VUS Gene** | **P/LP Gene** | **Gene-Elusive Overlap Syndrome** | **VUS cDNA** | **VUS AA** | **Notable Family History** | **Notable Findings from Phenotypic Evaluation** |
| --- | --- | --- | --- | --- | --- | --- | --- |
| **1** | *KCNA5* | *TTN* |  | c.1327A>G | p.Ille443Val | Unremarkable | Ventricular couplets on ambulatory monitor |
| **2** | *KCNQ1* | *TTN* |  | c.1092C>G | p.Phe364Leu | Familial AF | Ventricular couplets on ambulatory monitor |
| **3** | *MYH6* | *TTN* |  | c.3382C>T | p.Arg1128Cys | HCM | LGE on CMR |
| **4** | *TTN* | *TTN* |  | c.70282 G>T | p.Val23428Leu | Familial AF, Heart Transplant | LGE, mildly reduced LV systolic function, NSVT |
| **5** | *TTN* | *TTN* |  | c.66617G>A | p.Cys22206Tyr | Familial AF | Unremarkable |
| **6** | *TTN* | *MYH7* |  | c.102790C>T | p.Leu34264Phe | Familial AF | NSVT on ambulatory monitor |
| **7** | *SCN5A* |  | DCM | c.5701G>A | p.Glu1901Lys | SUD | Mildly reduced LV systolic function |
| **8** | *TTN* |  | DCM | c.58870G>A | p.Asp19624Asn | PPM | Mildly reduced LV systolic function |
| **9** | *ACTC1* |  | ACM/NDLVC | c.309C>A | p.His103Gin | Unremarkable | LGE on CMR |
| **10** | *TRPM4* |  | ACM/NDLVC | c.2295dup | p.Arg766Alafs*193 | Unremarkable | Mildly reduced LV systolic function |
| **11** | *ABCC9* |  |  | c.1130T>C | p.Ile377Thr | Familial AF | Unremarkable |
| **12** | *ABCC9,*  *DES* |  |  | c.2408C>T, c.1023+6T>G | p.Thr803Ile, Intronic | Familial AF | NSVT on ambulatory monitor |
| **13** | *ACTC2* |  |  | c.1907 A>G | p.GLu636Gly | Familial AF, SUD | NSVT on ambulatory monitor |
| **14** | *ACTN2* |  |  | c.1490 C>T | p.Thr497Ile | Familial AF, Early PPM | Unremarkable |
| **15** | *ACTN2* |  |  | c.2161C>T | p.Arg721Cys | Unremarkable | Unremarkable |
| **16** | *CACNA1, TTN* |  |  | c.4140+5G>A, c.53969T>G | Intronic, p.Val17990Gly | SUD | Unremarkable |
| **17** | *DES* |  |  | c.727C>T | p.H243Y | SUD | LGE on CMR |
| **18** | *DES* |  |  | c.415 G>C | p.Glu139Gln | Unremarkable | Unremarkable |
| **19** | *DSP* |  |  | c.8324 C>T | p.Thr2775Ile | Familial AF | Mildly reduced LV systolic function |
| **20** | *DSP* |  |  | c.1419+5G>C | Intronic | HCM | LGE on CMR |
| **21** | *FLNC* |  |  | c.7155C>G | p.Ile2385Met | Unremarkable | Ventricular couplets on ambulatory monitor |
| **22** | *KCNH2, TTN* |  |  | c.865G>A, c.82220T>C | p.Glu289Lys, p.Ile27407Thr | SUD | LGE, NSVT on ambulatory monitor |
| **23** | *KCNJ2* |  |  | c.694 C>T | p.Leu232Phe | Familial AF | Unremarkable |
| **24** | *MYBPC3* |  |  | c.2500 C>T | p.Arg834Trp | Familial AF, Seizures, SUD | Unremarkable |
| **25** | *MYH6* |  |  | c.3808C>T | p.Arg1270Cys | Unremarkable | Ventricular couplets on ambulatory monitor |
| **26** | *MYH7* |  |  | C.3982G>A | p.Ala1328Thr | Unremarkable | Unremarkable |
| **27** | *PRKAG2* |  |  | c.1429 G>A | p.Asp477Asn | Unremarkable | Unremarkable |
| **28** | *RBM20* |  |  | c.2207A>C | p.Lys736Thr | Familial AF, SUD | LGE, NSVT on ambulatory monitor |
| **29** | *RYR2* |  |  | c.808C>T | p.His270Tyr | Unremarkable | Ventricular couplets on ambulatory monitor |
| **30** | *SCN5A* |  |  | c.1535C>T | p.Thr512Ile | Unremarkable | LGE on CMR |
| **31** | *TNNT2* |  |  | c.629A>T | p.Lys210Met | SUD | Unremarkable |
| **32** | *TPM1* |  |  | c.755G>T | p.Ser252Ile | SUD | LGE on CMR, mildly reduced LV systolic function |
| **33** | *TRPM4* |  |  | c.377G>C | p.Gly126Ala | Familial AF, ACM, SUD | Ventricular couplets on ambulatory monitor |
| **34** | *TTN* |  |  | c.60445 T>G | p.Tyr20149Asp | SUD | NSVT on ambulatory monitor |
| **35** | *TTN* |  |  | c.79547G>A | p.Gly26516Asp | Unremarkable | Unremarkable |
| **36** | *VCL* |  |  | c.622G>A | p.Ala208Thr | Familial AF | Mildly reduced LV systolic function, ventricular couplets on ambulatory monitor |

**SUPPLEMENTAL TABLE 5: Details of clinical testing**

|  | **Total**  **Cohort**  **(N=246*)** | **Genetic Evaluation Groups** | | |
| --- | --- | --- | --- | --- |
|  |  | **Positive**  **Genetic Testing**  **(N=52)** | **Gene Elusive AF Overlap**  **Syndrome**  **(N=22)** | **Negative**  **Genetic Evaluation**  **(N=172)** |
| 12-lead ECG at enrollment | 246 (100%) | 52 (100%) | 22 (100%) | 172 (100%) |
| Ambulatory monitor  None  24-48-hour Holter  7 to 30-day event monitor  Implanted device (ILR, PPM, ICD) | 37 (15%)  153 (62%)  49 (20%)  7 (3%) | 8 (15%)  36 (69%)  7 (14%)  1 (2%) | 6 (27%)  10 (46%)  5 (23%)  1 (5%) | 23 (13%)  107 (62%)  37 (22%)  5 (3%) |
| Cardiac imaging*  None  Cardiac MRI  Transthoracic echocardiogram | 16 (7%)  172 (70%)  154 (63%) | 2 (4%)  42 (81%)  33 (64%) | 1 (5%)  18 (82%)  15 (68%) | 13 (8%)  112 (65%)  106 (62%) |
| Exercise treadmill ECG | 112 (46%) | 34 (65%) | 5 (23%) | 73 (42%) |
| Sodium channel blocker challenge (procainamide) | 6 (2%) | 2 (4%) | 1 (5%) | 3 (2%) |
| *Participants may have had both a cardiac MRI and transthoracic echocardiogram | | | | |

**Supplemental Figure 4: Penetrance of the ventricular phenotype in probands.** Each box represents one proband with positive genetic testing (N=48). Similar to Figure 7, penetrance for a ventricular phenotype is high among DCM and ACM gene carriers. However, penetrance may be lower in variant carriers associated with HCM phenotypes, as probands showed higher penetrance (67%) compared to at-risk probands and family members (40%) based on 2 family members without an identified HCM phenotype.


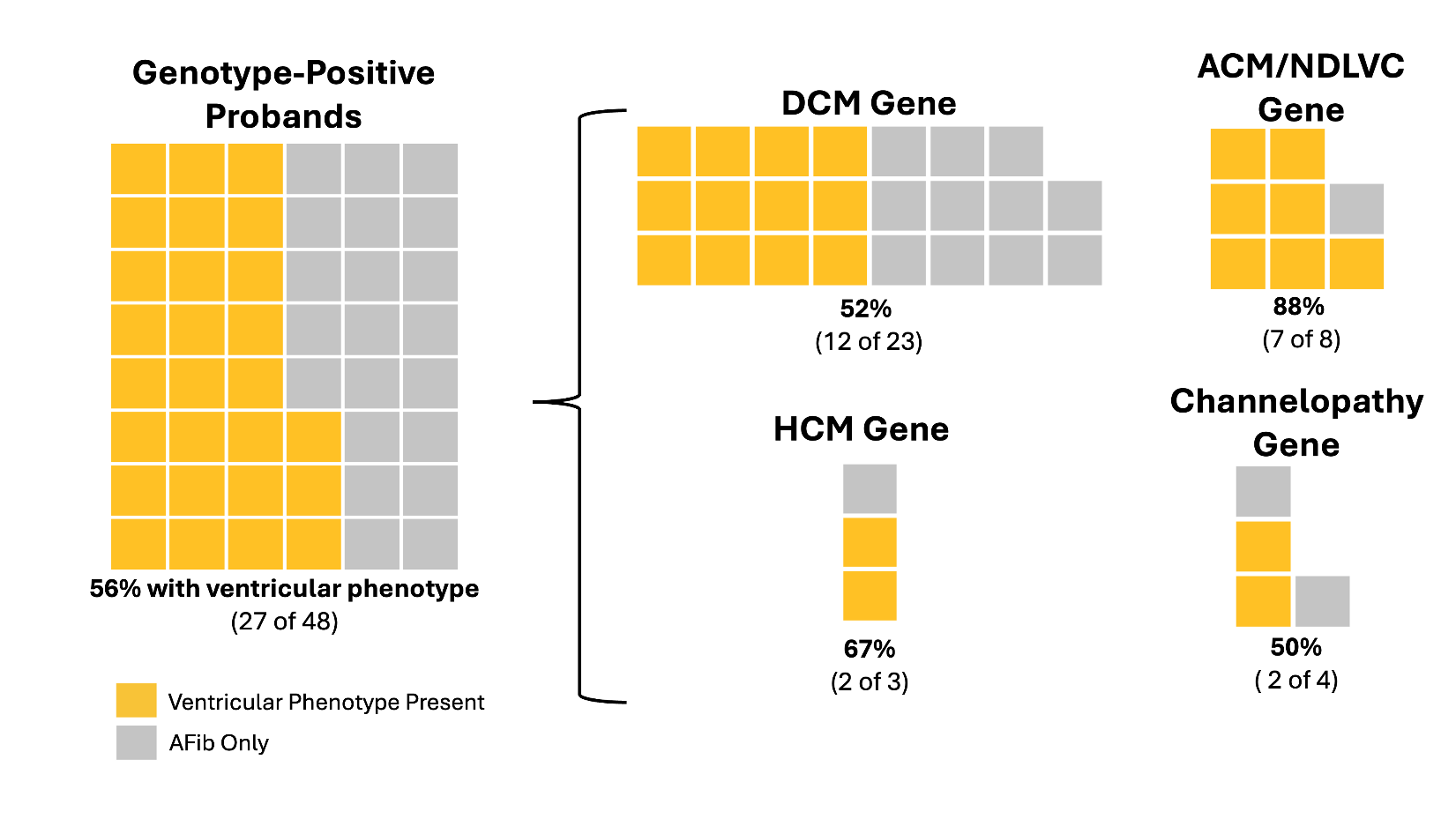


**Supplemental Table 6: Details of phenotype penetrance and changes to management**

|  | **Overall**  **(N=246)** | **Positive Genetic Testing**  **(N=52)** | **Gene-Elusive Overlap**  **(N=22)** |
| --- | --- | --- | --- |
| **Penetrance by Gene** | | | |
| Arrhythmogenic cardiomyopathy genes |  |  |  |
| Phenotype positive for ACM/NDLVC |  | 8 (89%) |  |
| Isolated AF |  | 1 (11%) |  |
| Dilated cardiomyopathy genes |  |  |  |
| Phenotype positive for DCM |  | 13 (54%) |  |
| Isolated AF |  | 11 (46%) |  |
| Hypertrophic cardiomyopathy genes |  |  |  |
| Phenotype positive for HCM |  | 2 (40%) |  |
| Isolated AF |  | 3 (60%) |  |
| Channelopathy genes |  |  |  |
| Phenotype positive for LQTS/Brugada/PCCD/CPVT |  | 2 (50%) |  |
| Isolated AF |  | 2 (50%) |  |
| Hemochromatosis (*HFE*) |  |  |  |
| Phenotype positive for Hemochromatosis |  | 2 (50%) |  |
| Isolated AF |  | 2 (50%) |  |
| Transthyretin amyloid (*TTR*) |  |  |  |
| Phenotype positive for Amyloidosis |  | 0 (0%) |  |
| Isolated AF |  | 2 (100%) |  |
| Genes with other predominant phenotype |  |  |  |
| Other phenotype positive |  | 2 (50%) |  |
| Isolated AF |  | 2 (50%) |  |
| **Management Changes** | | | |
| Any change to clinical management following AF Precision Medicine Clinic evaluation | 37 (15%) | 27 (52%) | 7 (32%) |
| Anticoagulation for stroke prophylaxis started | 7 (2.8%) | 4 (7.7%) | 3 (14%) |
| Beta-blocker started | 21 (8.5%) | 13 (25%) | 6 (27%) |
| ACEi/ARB started | 9 (3.7%) | 6 (12%) | 3 (14%) |
| Mineralocorticoid started | 3 (1.2%) | 3 (5.8%) | 0 (0%) |
| Neprilysin inhibitor started | 4 (1.6%) | 3 (5.8%) | 1 (4.5%) |
| SGLT2 inhibitor started | 7 (2.8%) | 6 (12%) | 1 (4.5%) |
| Physical activity recommendations | 9 (3.7%) | 7 (14%) | 1 (4.5%) |
| Antiarrhythmic drug precautions | 5 (2.0%) | 5 (9.6%) | 0 (0%) |
| New permanent pacemaker | 3 (1.2%) | 3 (5.8%) | 0 (0%) |
| New implantable cardioverter defibrillator | 7 (2.8%) | 7 (14%) | 0 (0%) |
| New implantable loop recorder (ILR) | 3 (1.2%) | 1 (1.9%) | 1 (4.5%) |
| EP study to evaluate VT/VF risk | 4 (1.6%) | 3 (5.8%) | 1 (4.5%) |
| Therapeutic phlebotomy started | 1 (0.4%) | 1 (1.9%) | 0 (0%) |
| For TTR amyloid, Tafamadis started | 0 (0%) | 0 (0%) | 0 (0%) |
| Cascade testing performed | 56 (23%) | 44 (85%) | 1 (4.5%) |

**Supplemental Table 7:** Summary statistics of regression models for clinical predictors of positive genetic testing

| **Clinical Predictor** | **OR** | **Lower 0.95** | **Upper 0.95** | **p-value** | **N/%** |
| --- | --- | --- | --- | --- | --- |
| Sustained VT/VF | 11.08 | 0.97 | 126.31 | 0.053 | 220/100 |
| History of cardiomyopathy | 5.26 | 2.09 | 13.27 | <0.0001 | 220/100 |
| History of non-sustained VT or couplets | 3.07 | 1.06 | 8.92 | 0.039 | 220/100 |
| BMI (25.0-29.9kg/m^2) | 2.51 | 0.84 | 7.46 | 0.098 | 220/100 |
| Diabetes mellitus | 2.28 | 0.86 | 6.08 | 0.099 | 220/100 |
| BMI (>=40kg/m^2) | 1.93 | 0.51 | 7.28 | 0.329 | 220/100 |
| Prior stroke or TIA | 1.81 | 0.40 | 8.17 | 0.441 | 220/100 |
| Permanent pacemaker | 1.61 | 0.33 | 7.74 | 0.555 | 220/100 |
| Permanent or persistent AF | 1.53 | 0.71 | 3.3 | 0.275 | 220/100 |
| History of PVCs | 1.51 | 0.61 | 3.72 | 0.375 | 220/100 |
| ≥3 cardioversions | 1.41 | 0.51 | 3.87 | 0.503 | 220/100 |
| BMI (30.0-39.9kg/m^2) | 1 | 0.33 | 2.99 | 0.996 | 220/100 |
| Smoking, vaping, or other tobacco | 0.85 | 0.39 | 1.81 | 0.668 | 220/100 |
| Thyroid disorder | 0.85 | 0.27 | 2.64 | 0.773 | 220/100 |
| Familial atrial fibrillation | 0.8 | 0.40 | 1.59 | 0.522 | 220/100 |
| Physical endurance training | 0.6 | 0.21 | 1.74 | 0.35 | 220/100 |
| Hypertension | 0.6 | 0.29 | 1.24 | 0.165 | 220/100 |
| Race (Non-White) | 0.52 | 0.19 | 1.44 | 0.209 | 220/100 |
| Syncope | 0.52 | 0.17 | 1.61 | 0.254 | 220/100 |
| Alcohol use disorder | 0.49 | 0.12 | 1.94 | 0.307 | 220/100 |
| Tall height (≥ 99th percentile) | 0.44 | 0.16 | 1.23 | 0.117 | 220/100 |
| Obstructive sleep apnea | 0.38 | 0.18 | 0.82 | 0.013 | 220/100 |
|  |  |  |  |  |  |
| Abnormal T1 | 4.04 | 1.45 | 11.22 | 0.007 | 121/55 |
| QTc >500 ms | 3.9 | 0.43 | 35.19 | 0.226 | 212/96 |
| Complex Ectopy (Couplets or NSVT) | 3.29 | 0.97 | 11.18 | 0.057 | 181/82 |
| Conduction Disease (QRS>120 ms) | 3.29 | 1.32 | 8.18 | 0.011 | 220/100 |
| Basal Septal Hypertrophy | 2.75 | 0.43 | 17.68 | 0.287 | 205/93 |
| QTc 470-499 ms | 2.49 | 0.56 | 11.2 | 0.233 | 212/96 |
| LGE on CMR | 2.44 | 1.00 | 5.97 | 0.05 | 148/67 |
| VE/day ≥500 | 2.4 | 0.89 | 6.46 | 0.084 | 179/81 |
| PR >= 200 ms | 2.28 | 0.79 | 6.57 | 0.126 | 198/90 |
| No Complex Ectopy | 2.27 | 0.68 | 7.53 | 0.18 | 181/82 |
| Low voltage QRS | 2.21 | 0.9 | 5.38 | 0.082 | 220/100 |
| Normal Cardiac Imaging | 1.98 | 0.84 | 4.65 | 0.118 | 220/100 |
| LV Dilation | 1.84 | 0.37 | 9.13 | 0.454 | 205/93 |
| Fascicular Block (QRS<120 ms) | 1.78 | 0.55 | 5.72 | 0.332 | 220/100 |
| VE/day 100-499 | 1.59 | 0.59 | 4.31 | 0.364 | 179/81 |
| LVEF <50% | 1.48 | 0.49 | 4.52 | 0.49 | 205/93 |
| LA Enlargement | 1.24 | 0.55 | 2.77 | 0.601 | 205/93 |
| RV Hypokinesis | 0.99 | 0.19 | 5.16 | 0.995 | 204/93 |
| Abnormal T2 | 0.87 | 0.09 | 8.45 | 0.901 | 121/55 |
| RV Dilation | 0.64 | 0.13 | 3.11 | 0.579 | 204/93 |
| HR < 60 bpm | 0.59 | 0.24 | 1.45 | 0.248 | 220/100 |
| Normal EKG | 0.49 | 0.25 | 0.97 | 0.042 | 220/100 |
| Concentric LVH | 0.39 | 0.08 | 1.79 | 0.225 | 205/93 |

**Supplemental References S.2**

1. Harris PA, Taylor R, Thielke R, Payne J, Gonzalez N, Conde JG. Research electronic data capture (REDCap)--a metadata-driven methodology and workflow process for providing translational research informatics support. J Biomed Inform. 2009 Apr;42(2):377–81.

2. DRAGEN sets new standard for data accuracy in PrecisionFDA benchmark data. Optimizing variant calling performance with Illumina machine learning and DRAGEN graph. [Internet]. [cited 2025 May 2]. Available from: https://www.illumina.com/content/illumina-marketing/amr/en_US/science/genomics-research/articles/dragen-shines-again-precisionfda-truth-challenge-v2.html

3. Nykamp K, Anderson M, Powers M, Garcia J, Herrera B, Ho YY, et al. Sherloc: a comprehensive refinement of the ACMG–AMP variant classification criteria. Genet Med. 2017 Oct;19(10):1105–17.

4. Rehm HL, Alaimo JT, Aradhya S, Bayrak-Toydemir P, Best H, Brandon R, et al. The landscape of reported VUS in multi-gene panel and genomic testing: Time for a change. Genet Med [Internet]. 2023 Dec 1 [cited 2025 May 2];25(12). Available from: https://www.gimjournal.org/article/S1098-3600(23)00960-7/fulltext

5. Richards S, Aziz N, Bale S, Bick D, Das S, Gastier-Foster J, et al. Standards and guidelines for the interpretation of sequence variants: a joint consensus recommendation of the American College of Medical Genetics and Genomics and the Association for Molecular Pathology. Genet Med Off J Am Coll Med Genet. 2015 May;17(5):405–24.

6. Arbelo E, Protonotarios A, Gimeno JR, Arbustini E, Barriales-Villa R, Basso C, et al. 2023 ESC Guidelines for the management of cardiomyopathies. Eur Heart J. 2023 Oct 1;44(37):3503–626.

7. Heidenreich PA, Bozkurt B, Aguilar D, Allen LA, Byun JJ, Colvin MM, et al. 2022 AHA/ACC/HFSA Guideline for the Management of Heart Failure. J Am Coll Cardiol. 2022 May;79(17):e263–421.

8. Ommen SR, Ho CY, Asif IM, Balaji S, Burke MA, Day SM, et al. 2024 AHA/ACC/AMSSM/HRS/PACES/SCMR Guideline for the Management of Hypertrophic Cardiomyopathy: A Report of the American Heart Association/American College of Cardiology Joint Committee on Clinical Practice Guidelines. Circulation. 2024 Jun 4;149(23):e1239–311.

9. Marcus FI, McKenna WJ, Sherrill D, Basso C, Bauce B, Bluemke DA, et al. Diagnosis of arrhythmogenic right ventricular cardiomyopathy/dysplasia: Proposed Modification of the Task Force Criteria. Eur Heart J. 2010 Apr 1;31(7):806–14.

10. Towbin JA, McKenna WJ, Abrams DJ, Ackerman MJ, Calkins H, Darrieux FCC, et al. 2019 HRS expert consensus statement on evaluation, risk stratification, and management of arrhythmogenic cardiomyopathy. Heart Rhythm. 2019 Nov;16(11):e301–72.

11. Priori SG, Wilde AA, Horie M, Cho Y, Behr ER, Berul C, et al. HRS/EHRA/APHRS expert consensus statement on the diagnosis and management of patients with inherited primary arrhythmia syndromes: document endorsed by HRS, EHRA, and APHRS in May 2013 and by ACCF, AHA, PACES, and AEPC in June 2013. Heart Rhythm. 2013 Dec;10(12):1932–63.

12. Kowdley KV, Brown KE, Ahn J, Sundaram V. ACG Clinical Guideline: Hereditary Hemochromatosis. Off J Am Coll Gastroenterol ACG. 2019 Aug;114(8):1202.

13. Contributors and Hemochromatosis International Taskforce, Adams P, Altes A, Brissot P, Butzeck B, Cabantchik I, et al. Therapeutic recommendations in HFE hemochromatosis for p.Cys282Tyr (C282Y/C282Y) homozygous genotype. Hepatol Int. 2018 Mar;12(2):83–6.

14. Kittleson MM, Ruberg FL, Ambardekar AV, Brannagan TH, Cheng RK, Clarke JO, et al. 2023 ACC Expert Consensus Decision Pathway on Comprehensive Multidisciplinary Care for the Patient With Cardiac Amyloidosis: A Report of the American College of Cardiology Solution Set Oversight Committee. J Am Coll Cardiol. 2023 Mar 21;81(11):1076–126.
